# Development of a rapid LAMP assay toward point-of-care detection of dermatophyte infections and antifungal resistance

**DOI:** 10.1128/spectrum.00669-26

**Published:** 2026-09-24

**Authors:** Georgia Wunderlich, Shannen Butterly, Michael Johnson, Artiene Tatian, Jake Baum

**Affiliations:** 1 School of Biomedical Sciences, University of New South Wales 85087, Sydney, Australia; 2 St. Vincent's Centre for Applied Medical Research, Sydney, Australia; 3 School of Clinical Medicine, University of New South Wales, Kensington, NSW, Australia; 4 Department of Paediatric Dermatology, Sydney Children's Hospital Randwick 63623, Randwick, NSW, Australia; 5 Department of Life Sciences, Imperial College London, London, United Kingdom; Memorial Sloan Kettering Cancer Center, New York, New York, USA; Mycology Laboratory, Wadsworth center, Albany, New York, USA; The Hong Kong Polytechnic University, Hong Kong, Hong Kong

**Keywords:** squalene epoxidase (SQLE) gene, single-nucleotide polymorphism (SNP), point-of-care diagnostic, fungal priority pathogens

## Abstract

Dermatophytosis is a common fungal infection affecting up to 25% of the global population. Increasing antifungal resistance and limited access to timely diagnostics hamper effective management, particularly in resource-limited settings. There is therefore an urgent need for reliable point-of-care diagnostics to guide treatment and support antifungal stewardship. Here, we report the development and validation of molecular assays based on loop-mediated isothermal amplification (LAMP) for rapid identification of dermatophytes and antifungal resistance-associated alleles. For taxonomic identification, we developed conventional LAMP assays targeting pan-dermatophytes, *Microsporum* spp., *Trichophyton rubrum* complex, and *Trichophyton mentagrophytes* complex. For detection of single-nucleotide polymorphisms (SNPs) associated with terbinafine resistance, we developed competitive SNP-based LAMP (sbLAMP) assays using unmodified self-stabilizing (USS) primers to differentiate specific from non-specific amplification. At 65°C, pan-dermatophyte, *Microsporum* spp., *T. rubrum* complex, and *T. mentagrophytes* complex samples were detected in less than 9 min of assay start, with *Microsporum* detection possible in less than 6 min. Limits of detection were 10 gene copies per reaction for *T. mentagrophytes* and 100–1,000 gene copies per reaction for the remaining assays. Using available clinical isolates, assays demonstrated sensitive and specific detection and were compatible with a simple colorimetric readout. For resistance detection, optimized primers validated wild-type allelic status of *T. rubrum* isolates, showing delayed amplification in mutant assays. Together, these assays show a means by which taxonomic identification and allele-specific resistance detection of dermatophyte infections might be possible within a point-of-care application. Such an application could support targeted treatment in remote or resource-limited settings that will also aid in the stewardship of emerging antifungal resistance.

**IMPORTANCE:**

Antifungal resistance is an emerging global health threat, with the World Health Organization identifying inadequate fungal diagnostics as a major barrier to effective treatment and surveillance. Dermatophyte infections affect up to one-quarter of the global population, yet diagnosis remains slow, laboratory-dependent, and often inaccessible in the communities most affected. This diagnostic gap contributes to empirical antifungal use, delayed treatment, and the emergence of resistance to frontline therapies, such as terbinafine. Here, we present a proof-of-concept molecular diagnostic platform based on loop-mediated isothermal amplification (LAMP) for the rapid identification of dermatophyte infections and associated antifungal resistance markers. By integrating species-level detection with allele-specific resistance profiling in a rapid, low-cost, and portable format, these assays provide a foundation for future point-of-care diagnostics that could improve access to timely fungal diagnosis, enable targeted antifungal therapy, and strengthen global antifungal stewardship, particularly in resource-limited settings.

## INTRODUCTION

Dermatophytosis, commonly known as ringworm or tinea, is a widespread fungal infection of the skin caused by a group of keratinophilic fungi collectively known as dermatophytes. The main causative agents belong to the genera *Trichophyton* and *Microsporum,* which differ in host site specificity and geographical distribution and can be geophilic, zoophilic, or anthropophilic in origin (1). Increasingly, the global burden of fungal disease is being compounded by the emergence of novel fungal pathogens and rising antifungal resistance, underappreciated consequences of global warming and widespread antifungal use (2). Despite their substantial global burden, fungal infections receive limited attention in public health frameworks, and current diagnostic approaches are frequently inadequate for timely and accessible pathogen identification, especially in remote and resource-limited settings, where access to laboratory infrastructure and specialist expertise is restricted.

The clinical features of dermatophytosis, such as chronic itching and skin scaling, can vary greatly between cases and can be mistaken for other dermatological diseases, such as psoriasis, eczema, or contact dermatitis. Currently, diagnosis relies on clinical evaluation and lab tests, including microscopy, culture, and histopathology (3). However, these conventional diagnostic methods are limited by prolonged turnaround times, often exceeding 4 weeks, challenges in species-level identification, and high rates of culture negativity. Consequently, antifungal therapy is commonly initiated empirically, contributing to inappropriate antifungal use. For example, in a recent evaluation of antifungal prescribing practices at a tertiary care institution in Spain, empirical treatment accounted for a substantial proportion of antifungal prescriptions (42%) (4). Optimization of antifungal use was estimated to result in significant cost savings, underscoring the clinical and economic impact of improved antifungal stewardship (4).

Compounding these challenges is the emergence and expansion of resistance to antifungal drugs, including terbinafine, the first-line treatment for dermatophytosis. The global spread of terbinafine-resistant dermatophytes represents a growing public health concern. A study of 402 patients in India found that 76% of *Trichophyton mentagrophytes* complex isolates from Northern India were resistant to terbinafine (5). More recently, widespread dissemination of the primary causative species *Trichophyton indotineae* has been reported across multiple continents, including Europe, Asia, North America, the Middle East, and Australia (6). Resistance is most commonly associated with single-nucleotide polymorphisms (SNPs) in the squalene epoxidase (SQLE) gene, which impair terbinafine activity (5). The rapid rise in treatment-resistant fungal infections prompted the World Health Organization to publish its first comprehensive assessment highlighting critical gaps in the availability of diagnostics for fungal priority pathogens (7).

Point-of-care diagnosis for infectious diseases is widely recognized as having great potential to dramatically change both clinical practice and improve patient outcomes. Although molecular diagnostics offer high sensitivity and specificity for fungal detection, their reliance on centralized laboratory infrastructure limits accessibility in many high-burden settings, underscoring the need for rapid, field-deployable diagnostic tools capable of supporting timely, evidence-based treatment decisions at the point-of-care. Diagnostics based on nucleic acid amplification are often seen as the gold standard for definitive pathogen identification. Beyond polymerase chain reaction (PCR), loop-mediated isothermal amplification (LAMP) has emerged as a promising alternative for rapid pathogen detection. LAMP enables specific DNA amplification but does so under isothermal conditions (8). These conditions eliminate the need for thermocycling and allow for faster turnaround and simplified equipment requirements. Advanced LAMP variants, such as unmodified self-stabilizing competitive primer-based SNP LAMP (USS-sbLAMP), further enable single-nucleotide mismatch discrimination and are therefore well suited for detection of resistance-associated mutations (9).

To aid in the diagnosis and monitoring of dermatophytosis infections, here we sought to develop and validate rapid, low-cost molecular diagnostic assays based on LAMP that could in the future be used at point-of-care. Given the rapidly evolving landscape of dermatophyte antifungal resistance, including the emergence of *T. indotineae*, this study was designed as a proof-of-concept framework that can be adapted to additional species and resistance-associated mutations as they emerge. The resulting platform would then enable simultaneous detection of dermatophyte species and clinically relevant antifungal resistance mutations directly from clinical samples, supporting improved treatment decisions and surveillance of antifungal resistance.

## MATERIALS AND METHODS

### Dermatophyte LAMP primer design

LAMP primer sets were selected or designed for four clinically relevant dermatophyte targets. The *Microsporum* assay was designed using the software Primer Explorer version 5.0 (Eiken Chemical Co. Ltd., Tokyo, Japan; http://primerexplorer.jp/lampv5e/index.html) based on reference sequences of selected genes retrieved from the NCBI GenBank database (https://www.ncbi.nlm.nih.gov/genbank/). Sequences were aligned using the MUSCLE algorithm (10) in Geneious Prime version 2025.1.2 (11) to identify conserved, species-specific regions suitable for primer design. Multiple primer sets were generated through Primer Explorer, with a single set selected based on shortest time-to-positive (TTP). The pan-dermatophyte primer set was adapted from Roumani et al. (12) with base modifications introduced to enhance specificity for members of the *Arthrodermataceae* family, which includes the nine recognized dermatophyte genera. Primer sets targeting the *Trichophyton rubrum* complex and the *Trichophyton mentagrophytes* complex were adopted directly from previously published studies (see Table 1) and were re-evaluated using the primer mapping function in Geneious Prime to confirm specificity and assess for potential cross-reactivity. All primers were purchased from Integrated DNA Technologies (IDT) and rehydrated in Tris-EDTA (TE) buffer at 100 μM. Primer sequences are shown in Table S1. Synthetic gene templates for target genes were ordered directly from IDT or TWIST Biosciences (synthetic gene template sequences in Table S2).

**TABLE 1:** Summary of taxonomic-based assays used in this work^*a*^

Assay	Limit of detection (copies/reaction)	Gene	Gene abbreviation	Minimum TTP (min)	References
Pan-dermatophyte	1,000	18S rRNA gene	18S	8.36	This study
*Microsporum* species	1,000	Translation elongation factor 1 alpha	Tef1α	5.85	This study
*Trichophyton rubrum* complex	100	Internal transcribed spacer region 1	ITS1	8.83	(13)
*Trichophyton mentagrophytes* complex	10	Internal transcribed spacer region 1	ITS1	7.10	(14)

^*a*^ Minimum TTP at 1 × 10^6^ copies/reaction (synthetic template).

### USS-sb LAMP primer design

For specific SNP detection, mutant (MT) and wild-type (WT) reactions were performed as described in Malpartida-Cardenas et al. (9). These reactions consisted of SNP-based LAMP (sbLAMP) primers targeting the specific allele for amplification (i.e., MT allele), and unmodified self-stabilizing (USS) competitive primers targeting the other allele (i.e., WT allele) to robustly delay or prevent unspecific amplification. Sequences (*n* = 368) of the SQLE gene from different *Trichophyton* species were aligned using the MUSCLE algorithm in Geneious Prime, and the documented mutation L393F was mapped on aligned sequences. The amino acid mutation was found to be caused by one of two base pair changes: TTT (herein MT) or TTC (herein MT-2); WT allele = TTA. All sbLAMP primer sets were made using Primer Explorer V5 and optimized manually to ensure the SNP of interest was located at the 5′ end of both F1c and B1c of the inner primers, following instructions from Eiken Chemical Co., Ltd. (https://primerexplorer.eiken.co.jp/e/v5_manual/index.html, accessed 16/06/2025). Varying lengths of F1c and B1c were designed for each SNP (17, 19, 21, 23 bp of F1c and 21, 23, 25, 27 bp of B1c). The USS primer sets were designed based on the most suitable pair of F1c and B1c for allele-specific amplification based on experimental data (see Results and Discussion). FB and BB primers of different lengths were manually designed, always equal to or longer than the selected F1c and B1c. Sequences of primers can be found in Table S1. Each USS-sbLAMP reaction followed the usual LAMP reaction protocol with the inclusion of 10 additional cycles.

### LAMP reaction conditions

Each LAMP reaction (5 μL total volume) contained the following components: 0.75 μL nuclease-free water, 0.5 μL 10× isothermal buffer (New England Biolabs), 0.3 μL MgSO₄ (100 mM), 0.7 μL dNTPs (10 mM), 0.125 μL bovine serum albumin (20 mg/mL), 0.8 μL betaine (5 M), 0.125 μL SYTO-9 Green (20 μM), 0.2 μL Bst 2.0 DNA polymerase (8,000 U/mL), 0.5 μL 10× LAMP primer mix (containing 20 μM BIP/FIP, 10 μM LF/LB, and 2.5 μM B3/F3), and 1 μL of template DNA (synthetic DNA for assay optimization and analytical sensitivity testing, or genomic DNA where indicated). Following temperature optimization, 65°C was determined to be the optimal temperature for simultaneous amplification across all assays. Therefore, all reactions were performed at 65°C for 40 min, followed by a melt curve analysis from 65°C to 97°C at a rate of 0.1°C/s to validate the specificity of the amplification products. Experiments were conducted in triplicate using the Bio-Rad CFX96 real-time PCR system (Bio-Rad, USA), with fluorescence detection in the FAM channel (excitation: 485 nm; emission: 498 nm).

### Samples and DNA extraction methods

Ten clinical dermatophyte isolates were obtained from Douglass Hanly Moir Pathology (Sydney, Australia). The isolates were provided as mycelial material stored in Microbank cryovials (Pro-Lab Diagnostics, Canada). An additional 11 yeast isolates were also tested for broader fungal cross-reactivity (details in Table S3). For DNA extraction, up to 250 mg of mycelia was transferred directly into microcentrifuge tubes. DNA was extracted using the DNeasy PowerSoil Pro Kit (QIAGEN, Germany) according to the manufacturer’s instructions. DNA was quantified using the Qubit fluorometer (Invitrogen, United States). To validate downstream molecular use, all samples underwent PCR amplification of the internal transcribed spacer (ITS) gene. PCR sample reactions (25 µL) included 12.5 µL DreamTaq PCR Master Mix (2X) (Thermofisher), 1 µL ITS1 primer (5 mM), 1 µL ITS4 primer (5 mM), 5.5 µL nuclease-free water, and 5 µL neat DNA. Thermocycler protocol: 95°C for 3 min, followed by 28 cycles of 95°C for 30 s, 52°C for 30 s, and 72°C for 1 min, with a final extension at 72°C for 5 min. Reactions were carried out in the T100 Thermal Cycler (Bio-Rad Laboratories, United States). Successful amplification was confirmed by visualization of banding patterns on agarose gel electrophoresis.

### Squalene epoxidase gene sequencing and analysis

Partial SQLE sequences of the clinical *Trichophyton* isolates were analyzed to determine allele phenotype. PCR sample reactions (25 µL) included 12.5 µL DreamTaq PCR Master Mix (2X) (Thermofisher), 1 µL TrSQLE-F1 (10 mM), 1 µL TrSQLE-R1 primer (10 mM), 9.5 µL nuclease-free water, and 1 µL neat DNA. Thermocycler protocol: 95°C for 3 min, followed by 30 cycles of 95°C for 1 min, 55°C for 30 s, and 72°C for 3 min, with a final extension at 72°C for 5 min (15). Successful amplification was checked by gel electrophoresis, and PCR products were purified using the MinElute PCR Purification Kit (QIAGEN, Germany) according to the manufacturer’s instructions. Purified PCR products were sent for bidirectional Sanger sequencing at the Ramaciotti Center for Genomics (UNSW Sydney, Australia). Nucleotide and predicted amino acid sequences obtained were compared with reference sequences available in NCBI to verify allele phenotype (WT, MT-1, or MT-2).

### Analytical sensitivity and specificity

Analytical sensitivity was evaluated using 10-fold serial dilutions of synthetic DNA ranging from 10⁷ to 10 copies/μL. Each dilution point was tested in triplicate on the Bio-Rad CFX96 real-time PCR system. Analytical specificity was assessed using the ten clinical dermatophyte isolates (see above). Isolates were identified using standard diagnostic procedures prior to inclusion.

### Colorimetric assay

To test compatibility of developed LAMP assays with a simple colorimetric output, assays were tested with the WarmStart Colorimetric LAMP 2X Master Mix (New England BioLabs). Reactions (10 μL) contained 5 μL WarmStart Colorimetric LAMP 2X Master Mix (New England BioLabs), 2.5 μL 10× LAMP primer mix as previously described, 2 μL neat DNA, and μL water. Reactions were set up at room temperature and incubated at 65°C in the T100 Thermal Cycler. Reactions were checked at 5 min intervals (up to 40 min) for signs of a color shift from pink (negative) to positive (yellow), indicating amplification has taken place.

### Statistical analysis

TTP results are reported as the mean ± standard deviation. A semilogarithmic linear regression was performed in GraphPad Prism (version 10.6.1) to model the relationship between copy number and TTP. Statistical comparisons were calculated using Student’s heteroscedastic *t*-test, with a two-sided distribution. Statistically significant differences were considered as *P value < 0.05, **P value < 0.01, ***P value < 0.001.

## RESULTS

### Analytical sensitivity and specificity of LAMP assays

LAMP primer sets were selected or designed to discriminate four clinically relevant dermatophyte targets: pan-dermatophyte, *Microsporum* spp., *T. rubrum* complex (13), and *T. mentagrophytes* complex (14). Pan-dermatophyte and *Microsporum* assay primer sets were designed using the software Primer Explorer version 5.0 (http://primerexplorer.jp/lampv5e/index.html) based on reference sequences retrieved from the NCBI GenBank database (https://www.ncbi.nlm.nih.gov/genbank/). The pan-dermatophyte assay was designed to target all members of the *Arthrodermataceae* family, which includes the nine recognized dermatophyte genera. Based on multi-sequence alignment of several taxonomic marker genes (Tef-1α, ITS, D1/D2, and 18S), the 18S locus was selected for the pan-dermatophyte assay due to its conservation across dermatophytes but divergence from other fungal families, and similarly, the Tef-1α for *Microsporum* spp. Analytical specificity was initially assessed *in silico* based on multi-sequence alignment (MUSCLE algorithm), with primer sets shown to be highly specific with no cross-reactivity to non-target fungal species (minimum mismatch 2 bp per primer).

**Fig 1:**
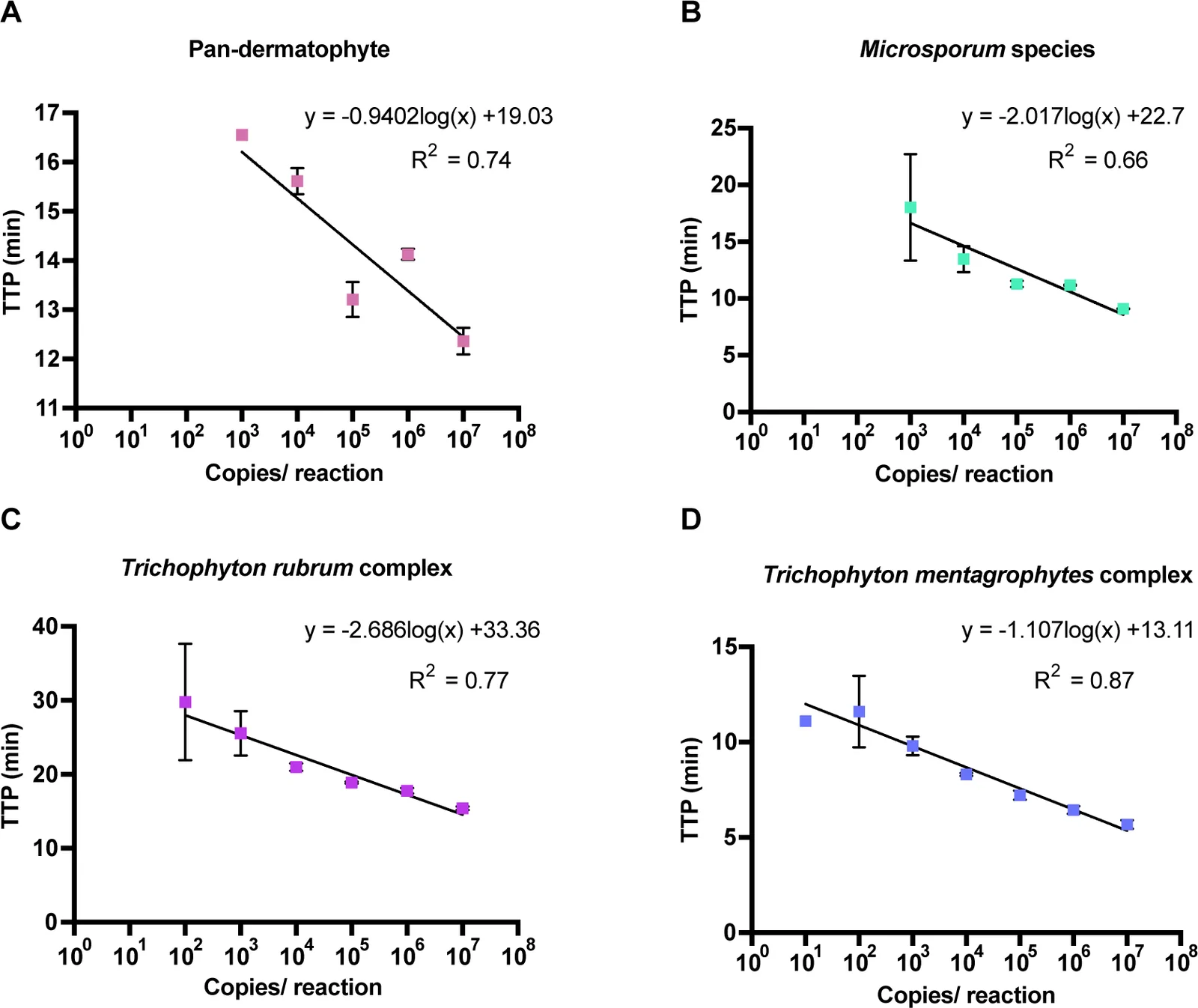
Analytical sensitivity of taxonomic-based LAMP assays. (**A**) Pan-dermatophyte standard curve, (**B**) *Microsporum* standard curve, (**C**) *T. rubrum* standard curve, and (**D**) *T. mentagrophytes* standard curve. For pan-dermatophyte assay, reactions were considered negative above 30 min and above 35 min for all other assays.

Synthetic gene templates were used to optimize and assess sensitivity and specificity via real-time PCR (following reference 16) (Fig. 1). Using a real-time PCR platform to allow visualization of TTP and amplification specificity, all assays could be shown to amplify target DNA in under 30 min (Table 1; Fig. 1A through D). The *T. mentagrophytes* assay showed the highest sensitivity, detecting as few as 10 copies/reaction (Table 1; Fig. 1D). All other assays had a limit of detection of 100–1,000 copies/reaction (Table 1; Fig. 1A through C). The pan-dermatophyte, *Microsporum,* and *T. rubrum* assays variably showed non-specific amplification in the non-template control (H_2_O) beyond 30 min (example amplification plot and melt curve in Fig. S1). As such, in subsequent assays, we capped running times of the pan-dermatophyte assay to 30 min and all other assays to 35 min, in line with previously developed LAMP assays (16).

**Fig 2:**
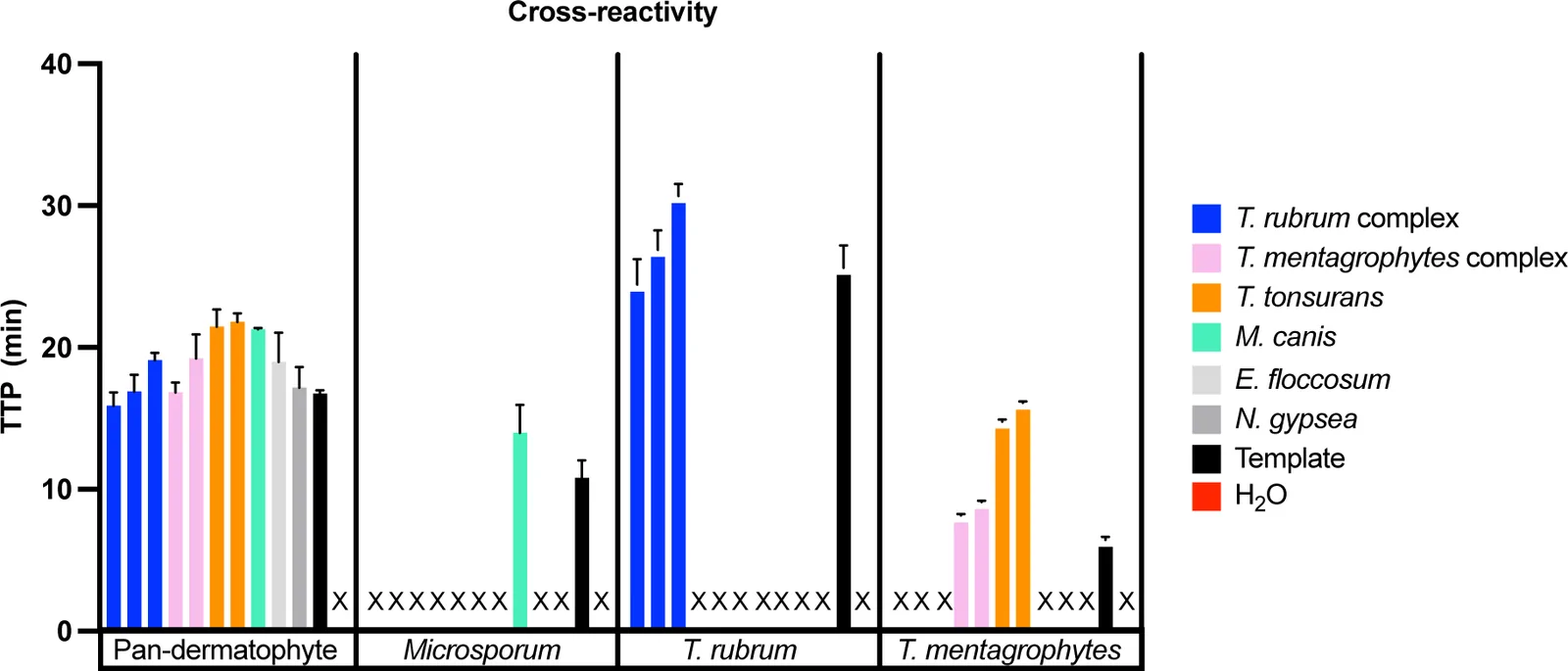
Cross-reactivity of taxonomic-based LAMP assays using clinical isolates (*n* = 10). Assay-specific synthetic templates (black bars) were used as positive controls and H_2_O as a negative control. For pan-dermatophyte assay, reactions were considered negative above 30 min and above 35 min for all other assays.

To validate assays with authentic DNA samples, clinical isolates from dermatophytosis-infected tissue were obtained (*n* = 10) to validate and assess cross-reactivity with all four taxonomic assays (Fig. 2). Respective synthetic gene templates were used as positive controls for each assay and H_2_O as a non-template control. The pan-dermatophyte assay successfully amplified all dermatophyte clinical isolates (10/10). The *Microsporum* and *T. rubrum* assays only amplified samples containing their targeted organisms. The *T. mentagrophytes* assay amplified target complex organisms and delayed amplification of *Trichophyton tonsurans*.

### Colorimetric validation of taxonomic assay

**Fig 3:**
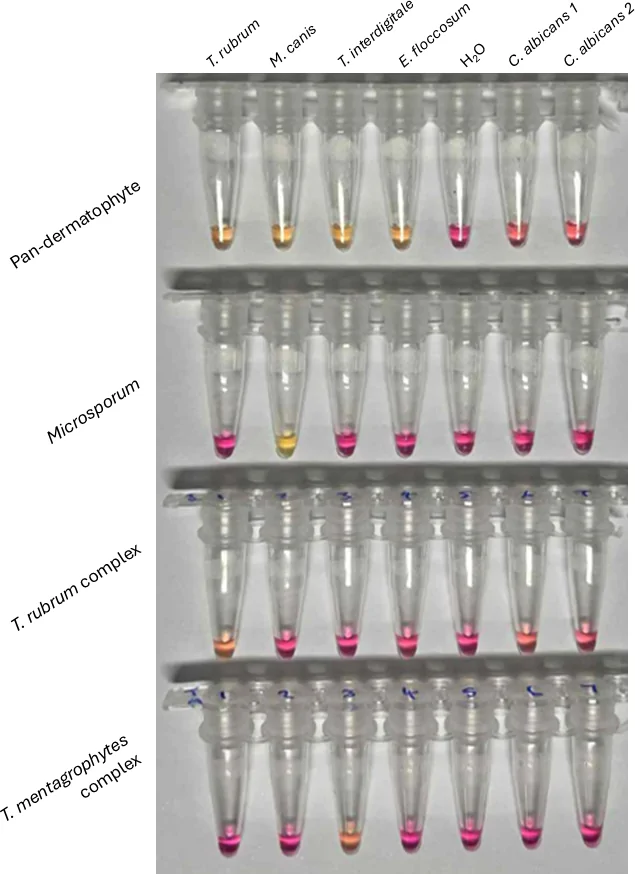
Colorimetric validation of developed LAMP assays for dermatophyte taxonomic LAMP assays. The four developed assays were tested with four clinical dermatophyte isolates, H_2_O as a negative control, and two additional negative controls containing clinical *Candida albicans* isolates. The assay results shown were visualized after an incubation of 30 min.

Taxonomic assays were then tested for compatibility with a simple colorimetric readout by incubating them in a thermocycler and checking every 5 min for color change, indicating amplification. All four taxonomic-based assays successfully amplified target species (Fig. 3). *Candida albicans* was used as a negative control fungal isolate to test for specificity to dermatophyte species. These samples had some color change after 30 min for both the *T. rubrum* and pan-dermatophyte assays but not earlier.

### Development of sbLAMP assays

**Fig 4:**
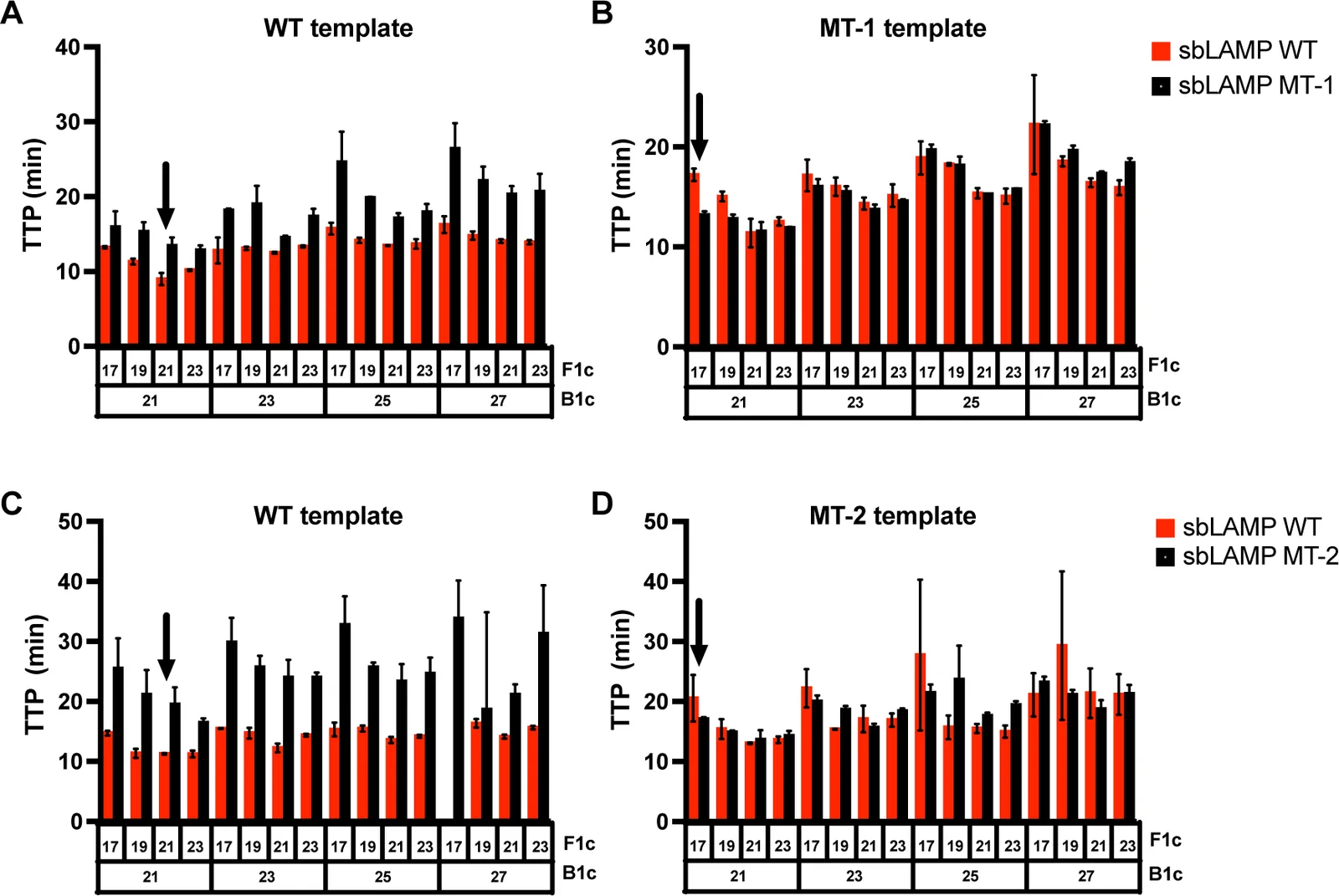
Results of sbLAMP using different F1c-B1c primer lengths. (**A**) Comparison of WT template amplification by WT-specific (sbLAMP_WT_) and MT-1-specific (sbLAMP_MT-1_) primer sets with different lengths of F1c and B1c. (**B**) Comparison of MT-1 template amplification by WT-specific (sbLAMP_WT_) and MT-1-specific (sbLAMP_MT-1_) primer sets with different lengths of F1c and B1c. (**C**) Comparison of WT template amplification by WT-specific (sbLAMP_WT_) and MT-2-specific (sbLAMP_MT-2_) primer sets with different lengths of F1c and B1c. (**D**) Comparison of MT-2 template amplification by WT-specific (sbLAMP_WT_) and MT-2-specific (sbLAMP_MT-2_) primer sets with different lengths of F1c and B1c. Selected sbLAMP primer sets are indicated by arrows.

To explore the compatibility of our LAMP-based assay platform with terbinafine resistance, we selected a SNP for sbLAMP-specific development, the L393F substitution of the SQLE gene of *T. rubrum*. While several SNPs are reported in the literature to pertain to terbinafine resistance, L393F was selected as a proof-of-concept mutation in this study due to its more frequent presence in resistance strains of *T. rubrum* and higher associated minimum inhibitory concentrations (17). Multiple primer pairs were developed to aid targeting of the SNP along with different lengths of F1c and B1c primers. Varying lengths were designed to study the impact of local GC% content, Tm, and primer length in (i) allele-specificity and (ii) TTP (following reference 9). Real-time amplification experiments of WT and MT templates were performed independently, each with two separate reactions (WT specific and MT/MT-2 specific). A total of 16 combinations of F1c-B1c were performed (Fig. 4). The amplification time difference (ΔTTP) between these reactions was calculated, and final sbLAMP primer sets were selected according to the highest ΔTTP (indicated by arrows in Fig. 4). WT template had the highest ΔTTP with F1c21 and B1c21 for both alleles (4.46 and 8.23 min, respectively ). Both MT templates (MT and MT-2) performed best with F1c17 and B1c21 (3.99 and 3.48 min, respectively) (Table 2). Detailed TTP values of all reactions are reported in Tables S4 and S5.

**TABLE 2:** Table showing the ΔTTP values between specific and non-specific primer sets (mean ΔTTP ± standard deviation)^*a*^

Reaction	F1C	B1C			
		17	19	21	23
Mutant 1 (TTT)	21	*3.99 ± 0.71	2.22 ± 0.64	−0.18 ± 1.69	0.63 ± 0.43
	23	1.1 ± 1.75	0.51 ± 1.06	0.6 ± 0.79	0.55 ± 1.14
	25	−0.78 ± 1.74	0.1 ± 0.89	0.08 ± 0.51	−0.7 ± 0.77
	27	0.05 ± 4.96	−1.08 ± 0.71	−0.91 ± 0.49	−2.48 ± 0.87
Mutant 2 (TTC)	21	*3.48 ± 3.9	0.51 ± 1.67	−0.64 ± 1.52	−0.66 ± 0.94
	23	2.2 ± 3.33	−3.31 ± 0.59	1.39 ± 2.28	−1.53 ± 1.19
	25	6.26 ± 12.66	−7.99 ± 5.93	−2.12 ± 0.91	−4.46 ± 1.19
	27	−2.06 ± 3.72	8.17 ± 12.39	2.6 ± 4.37	−0.12 ± 3.68

^*a*^ Selected sbLAMP primer sets are highlighted in bold/asterisks.

### Incorporation of USS primers for enhancement of sbLAMP

**Fig 5:**
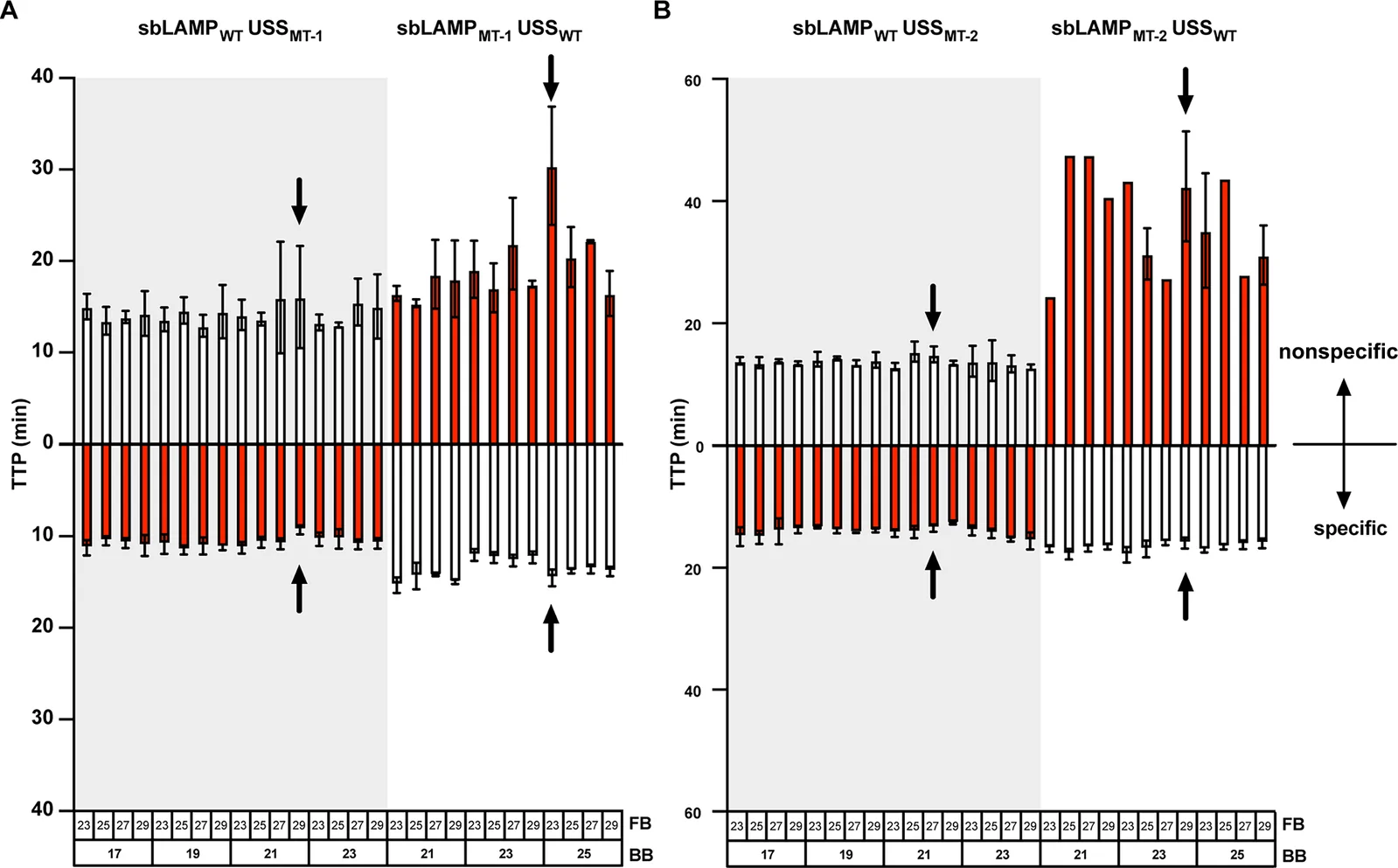
Performance comparison of USS primers with different lengths added to sbLAMP reaction for detection of SQLE mutations. (**A**) WT vs MT-1 reaction, (**B**) WT vs MT-2 reaction. TTP of specific reactions is shown in the lower sections, and TTP of non-specific reactions is in the upper section. Red bars represent amplification of the WT template, and white bars represent amplification of the corresponding MT template. In each panel, the grey-shaded region shows results obtained using the WT assay, and the unshaded region shows results obtained using the corresponding MT-specific assay. Selected USS primers are indicated with arrows.

USS primers were developed based on the optimal sbLAMP primer sets selected and were incorporated into the reaction at 2 µM to prevent unspecific sbLAMP amplification. FB and BB of different lengths were designed and tested, totaling 15 different combinations (Fig. 5). Again, two independent reactions were tested for each template: a WT reaction with mutant blocking primers (sbLAMP_WT_ and USS_MT/ MT-2_) and a MT reaction (sbLAMP_MT/ MT-2_ and USS_WT_). Detailed TTP values can be found in Tables S6 and S7. Selection of the optimal FB/BB primer length was based on minimizing the TTP of specific reactions while simultaneously maximizing the TTP of unspecific reactions. The selected lengths were USS_WT_ FB23 and BB25 for sbLAMP_MT_ reactions and USS_MT_ FB29 and BB21 for sbLAMP_WT_. For MT-2 reactions, the selected lengths were USS_WT_ FB29 and BB23 for sbLAMP_MT-2_ reactions and USS_MT-2_ FB27 and BB21 for sbLAMP_WT_.

### Clinical validation of USS-sbLAMP assays

**Fig 6:**
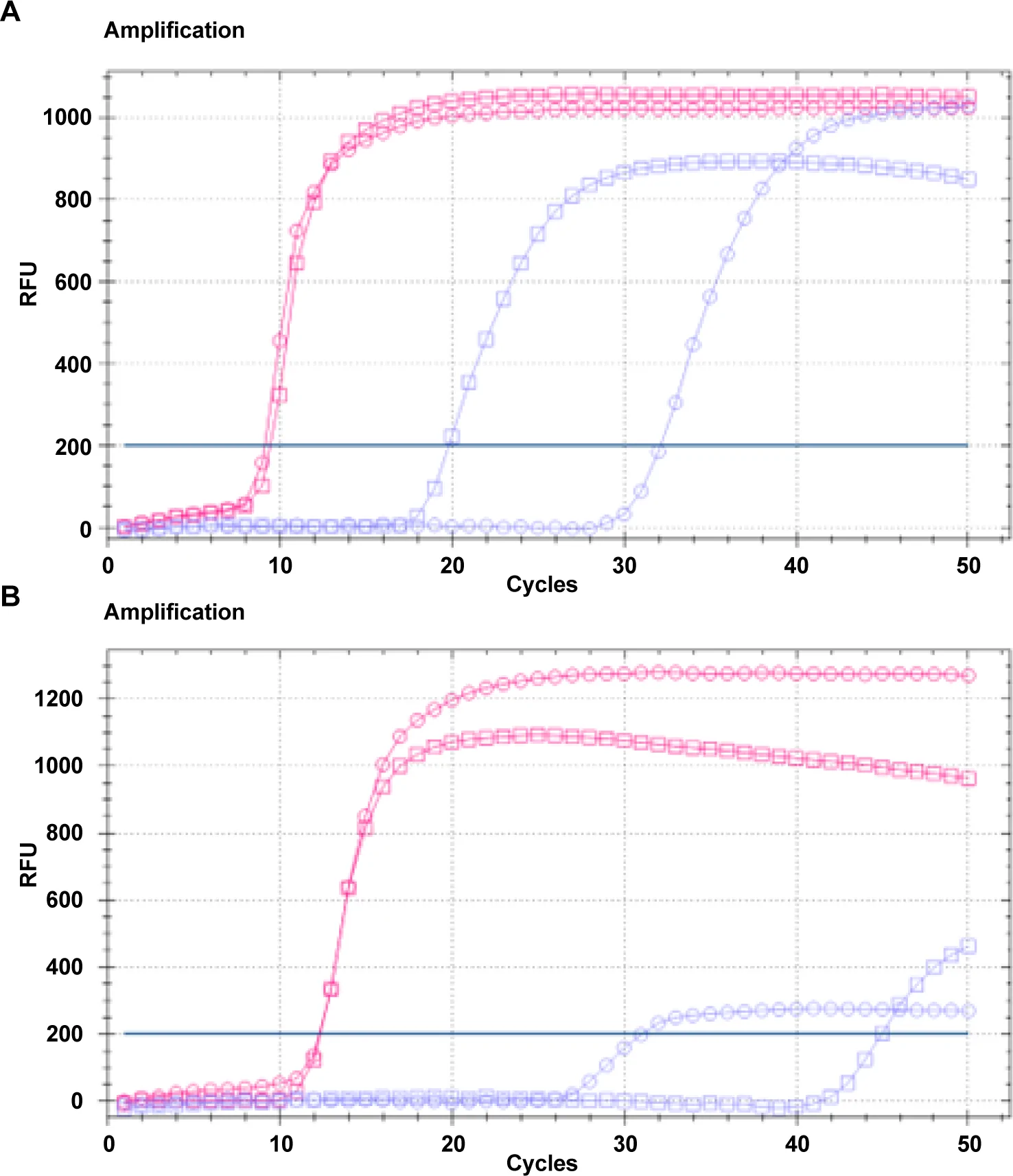
Real-time amplification plot of wild-type *Trichophyton rubrum* clinical isolates with USS-sbLAMP assays. (**A**) WT vs MT-1 reaction, (**B**) WT vs MT-2 reaction. Pink lines = WT reaction, blue lines = MT-1/-2 reaction. Fluorescence is plotted as relative fluorescence units (RFU) vs amplification cycles. Circles denote *T. rubrum* A clinical isolate, and squares denote *T. rubrum* B clinical isolate.

As with taxonomic assays, complete USS-sbLAMP assays were validated with clinical isolates using RT curve analysis (Table 3; Fig. 6). There was an expectation that clinical isolates may be enriched for resistance alleles (hence the progression of patient samples to clinic). To test this, and compare with assay prediction, the SQLE genes of each clinical *Trichophyton* species were sequenced to determine allele phenotype. Against expectations, *T. rubrum* A and B isolates were both found to possess the WT allele. Of note, WT *T. rubrum* isolates had delayed amplification with both MT assays, aligning with the allelic WT status as determined by sequencing. All TTPs for WT-specific assays were under 15 min, demonstrating the specificity of our sbLAMP assay and, combined with taxonomic status testing, demonstrating the feasibility of these approaches with future point-of-care diagnostic development.

**TABLE 3:** Performance of USS-sbLAMP assays with *Trichophyton rubrum* clinical isolates^*a*^

Clinical isolate	SQLE allele	sbLAMP_WT_ USS_MT-1_	sbLAMP_MT-1_ USS_WT_	ΔTTP (min)	sbLAMP_WT_ USS_MT-2_	sbLAMP_MT-2_ USS_WT_	ΔTTP (min)
*Trichophyton rubrum* (A)	WT	9.47 + 0.36	22.7 + 8.15	13.23 + 8.16	12.6 + 0.31	29.59 + 5.43	16.99 + 5.44
*Trichophyton rubrum* (B)	WT	10.06 + 0.62	32.22 + 11.58	22.16 + 11.6	12.22 + 0.17	37.07 + 11.06	24.85 + 11.06

^*a*^ Reactions performed in triplicate.

## DISCUSSION

In this study, we have developed and evaluated rapid, low-cost LAMP-based assays with potential for future point-of-care application toward detection of dermatophyte infections and terbinafine resistance. Taxonomic LAMP assays targeting pan-dermatophytes, *Microsporum* spp., *Trichophyton rubrum* complex, and *T. mentagrophytes* complex enabled sensitive and specific detection within 30 min, while allele-specific USS-sbLAMP assays discriminated clinically relevant *SQLE* resistance mutations. Together, these assays generate actionable diagnostic information within a single clinical encounter and represent a practical foundation for integration into portable LAMP or lab-on-chip diagnostic systems.

Dermatophytosis is estimated to affect up to 25% of the global population (18), with the burden greatest in tropical regions, remote communities, and resource-limited countries (e.g., regions of Africa/Asia), where access to mycology expertise and laboratory diagnostics is limited (1). In these contexts, prolonged diagnostic turnaround times increase the risk of ongoing transmission and recurrence and frequently lead to empirical antifungal prescribing (19), contributing to inappropriate drug use and selective pressure for resistance. Rapid, point-of-care diagnostics, such as those described here, may help mitigate these challenges by enabling earlier, evidence-based treatment decisions and reducing reliance on empirical therapy. LAMP-based point-of-care assays have been successfully applied to bacterial, fungal, viral, and parasitic pathogens, demonstrating their versatility for decentralized testing across diverse clinical contexts (16, 20–23).

All taxonomic assays developed demonstrated rapid amplification and high analytical sensitivity, with limits of detection ranging from 10 to 1,000 copies per reaction depending on the target (Fig. 1). These findings support the suitability of LAMP for dermatophyte detection in settings where fungal burden may be low and reaction inhibitors are common. Consistent with previous studies, the time to detection at lower concentrations was considerably more variable than at higher concentrations (24), indicating that time-to-positive or inferred copy number should not be interpreted as a reliable quantitative proxy for infectious burden. Low-level late amplification observed in some assays beyond defined runtimes highlights the importance of conservative assay cutoffs, which are compatible with point-of-care workflows prioritizing rapid decision-making.

Evaluation using available clinical isolates confirmed high specificity, with pan-dermatophyte primers detecting all dermatophyte isolates and species-complex assays amplifying only their intended targets (Fig. 2). A major strength of this assay is its compatibility with simple colorimetric readouts, enabling deployment without specialized instrumentation (Fig. 3). A similar approach has recently been applied successfully in the field for detection of Monkeypox infections (20). All taxonomic assays produced color changes within defined runtimes, supporting visual interpretation that is a prerequisite for many point-of-care applications. Notably, validation was performed using a limited number of clinical isolates and extracted genomic DNA rather than primary clinical specimens. Further validation using clinical samples (hair, skin, and nails) will be required to define diagnostic performance under routine use and suitable extraction conditions.

The L393F substitution in the SQLE gene was selected as a proof-of-concept resistance marker due to its strong association with elevated minimum inhibitory concentrations and treatment failure. In a study of 46 treatment-resistant dermatophytosis samples, 31% of these had the L393F mutation (17). To be considered clinically resistant to antifungal treatment, research has proposed an MIC of 0.015 μg/mL as a minimum breakpoint for predicting clinically relevant terbinafine treatment failure to standard oral dosing for dermatophyte infections. The L393F mutation has shown MICs ranging from 4 to 16 μg/mL.

Using USS-sbLAMP, we achieved robust discrimination between WT and MT SQLE alleles, including two nucleotide variants encoding L393F. Empirical optimization of primer architecture was essential to maximizing allele discrimination and minimizing non-specific amplification, underscoring the importance of experimental validation in SNP-targeted isothermal assays. Validation using WT clinical isolates demonstrated clear delayed amplification of isolates with the MT assays (Table 3), evident with visualization of RT amplification plots (Fig. 6). Future iterations will incorporate additional resistance-associated mutations to broaden clinical applicability. This is particularly relevant for the emerging pathogen *T. indotineae*, in which alternative SQLE mutations, such as L393S and F397L, are increasingly reported and represent logical future targets for assay expansion (5). While taxonomic LAMP assays were readily compatible with colorimetric detection, further refinement of the USS-sbLAMP method is needed to improve temporal discrimination between specific and non-specific reactions and allow a consistent and reliable color change.

In summary, here we present a proof-of-concept pathfinder for rapid, accessible LAMP-based assays for dermatophyte identification and antifungal resistance detection. The assay framework described here is readily adaptable to additional resistance-associated mutations and emerging dermatophyte species, supporting future diagnostic development in an evolving antifungal resistance landscape. By addressing key barriers to timely diagnosis and equitable access to diagnostics, these assays align with global priorities in antifungal stewardship and diagnostic equity and have the potential to improve outcomes for populations disproportionately affected by fungal skin infections.

## Supplementary Material

Reviewer comments

## Data Availability

All primer sequences are available in Table S1 and gene block sequences in Table S2. SQLE sequences of Trichophyton isolates were deposited into GenBank under accession numbers PZ843401–PZ843407.

## SUPPLEMENTAL MATERIAL

Supplemental figure and tables spectrum.00669-26-s0001.docx Fig. S1 and Tables S1 to S7.

OPEN PEER REVIEW reviewer-comments.pdf An accounting of the reviewer comments and feedback.

ASM does not own the copyrights to Supplemental Material that may be linked to, or accessed through, an article. The authors have granted ASM a non-exclusive, world-wide license to publish the Supplemental Material files. Please contact the corresponding author directly for reuse.
