# Supplementary material for: Development of a rapid LAMP assay toward point-of-care detection of dermatophyte infections and antifungal resistance
Source: Microbiol Spectr. 2026 Sep 24;14(11):e00669-26. doi: 10.1128/spectrum.00669-26 (PMC13729109; doi:10.1128/spectrum.00669-26)
Supplement: Supplementary file 1 [file reviewer-comments.pdf]

ASM | Microbiology Spectrum

Development of a Rapid LAMP Assay towards Point-of-Care Detection of Dermatophyte Infections and Antifungal Resistance

Georgia Wunderlich, Shannen Butterly, Michael Johnson, Artiene Tatian, and Jake Baum

Corresponding Author(s): Jake Baum, University of New South Wales School of Biomedical Sciences

Review Timeline:

Submission Date:	February 24, 2026
Editorial Decision:	May 23, 2026
Revision Received:	June 28, 2026
Editorial Decision:	August 2, 2026
Revision Received:	August 3, 2026
Accepted:	August 3, 2026

Editor: June Chan

Reviewer(s): Disclosure of reviewer identity is with reference to reviewer comments included in decision letter(s). The following individuals involved in review of your submission have agreed to reveal their identity: Sudha Chaturvedi (Reviewer #1); Franklin Wang-ngai Chow (Reviewer #2)

Transaction Report:

(Note: With the exception of the correction of typographical or spelling errors that could be a source of ambiguity, letters and reports are not edited. The original formatting of letters and referee reports may not be reflected in this compilation.)

DOI: https://doi.org/10.1128/spectrum.00669-26

Re: Spectrum00669-26 (Development of a Rapid LAMP Assay for Point-of-Care Detection of Dermatophyte Infections and Antifungal Resistance)

Dear Prof. Jake Baum:

Thank you for the privilege of reviewing your work. Below you will find my comments, instructions from the Spectrum editorial office, and the reviewer comments.

Please return the manuscript within 60 days; if you cannot complete the modification within this time period, please contact me. If you do not wish to modify the manuscript and prefer to submit it to another journal, notify me immediately so that the manuscript may be formally withdrawn from consideration by Spectrum.

Revision Guidelines
To submit your modified manuscript, log into the submission site at https://spectrum.msubmit.net/cgi-bin/main.plex. Go to Author Tasks and click the appropriate manuscript title to begin. The information you entered when you first submitted the paper will be displayed; update this as necessary. Note the following requirements:

• Upload point-by-point responses to the issues raised by the reviewers in a file named "Response to Reviewers," NOT in your cover letter.
• Upload a compare copy of the manuscript (without figures) as a "Marked-Up Manuscript" file.
• Upload a clean .DOC/.DOCX version of the revised manuscript and remove the previous version.
• Each figure must be uploaded as a separate, editable, high-resolution file (TIFF or EPS preferred), and any multipanel figures must be assembled into one file.
• Any supplemental material intended for posting by ASM should be uploaded with their legends separate from the main manuscript. You can combine all supplemental material into one file (preferred) or split it into a maximum of 10 files with all associated legends included.

For complete guidelines on revision requirements, see our Submission and Review Process webpage. Submission of a paper that does not conform to guidelines may delay acceptance of your manuscript.

Data availability: ASM policy requires that data be available to the public upon online posting of the article, so please verify all links to sequence records, if present, and make sure that each number retrieves the full record of the data. If a new accession number is not linked or a link is broken, provide Spectrum production staff with the correct URL for the record. If the accession numbers for new data are not publicly accessible before the expected online posting of the article, publication may be delayed; please contact production staff (Spectrum@asmusa.org) immediately with the expected release date.

Publication Fees: For information on publication fees and which article types are subject to charges, visit our website. If your manuscript is accepted for publication and any fees apply, you will be contacted separately about payment during the production process; please follow the instructions in that e-mail. Arrangements for payment must be made before your article is published.

ASM Membership: Corresponding authors may join or renew ASM membership to obtain discounts on publication fees. Need to upgrade your membership level? Please contact Customer Service at Service@asmusa.org.

The ASM Journals program strives for constant improvement in our submission and publication process. Please tell us how we can improve your experience by taking this quick Author Survey.

Thank you for submitting your paper to Spectrum.

Sincerely,
June Chan
Editor
Microbiology Spectrum

Reviewer #1 (Comments for the Author):

Authors have developed a LAMP assay for dermatophyte ID and terbinafine resistance as proof-of-concept experiments. The assay appears to be working, but without enough isolates, it is difficult to assess its potential. Authors should use more isolates, and also include T. mentagrophytes genotypes, especially TmVII and T. indotineae, as they are emerging pathogens. Also, assay sensitivity should be assessed with gDNA, not synthetic DNA. Additionally, assay specificity requires several isolates

within the species complex to test whether it works. Using only one isolate per complex is not sufficient, especially if this assay will be used for patient care. The authors used only 10 dermatophyte isolates for assay development, which is inadequate.
Line 129 - Authors talk about L393F mutation, but do not talk about mutations L393S and F397L, which appears to be major mutations causing resistance in T. indotineae. https://pubmed.ncbi.nlm.nih.gov/38748419/
Line 149 - Please clearly provide whether synthetic or gDNA used for which assay?
Line 224 - Authors have used synthetic DNA to determine the assay limit of detection. It is well known that synthetic DNA provides far greater sensitivity than genomic DNA due to its simpler structure compared to complex gDNA. Authors should use dermatophyte isolate gDNA for this study to better understand their assay potential, as that is what they will receive in the laboratory for both ID and resistance detection.
Line 233 - Why non template control showed amplification beyond 30 min. Please clarify
Line 247 - Unfortunately, 10 isolates are too small a number to determine assay validity. They should increase the number of total dermatophytes, especially for new assay. Also, why TTP values for template DNA (I believe that is control) with different dermatophytes section in Fig. 2 are different? Is template DNA is inhibiting?
Fig. 3. I don't see any of mentagrophytes or T. indotineae in this figure. Considering the emergence of T. mentagrophytes genotype VII, and drug-resistant T. indotineae, it would be good to have these two at least for part of this assay.
Fig. 6. Are MT-1 or MT-2 is synthetic construct or mutant strain of T. rubrum?

Reviewer #2 (Comments for the Author):

This manuscript addresses a clinically relevant and timely problem-the need for rapid point-of-care diagnostics for dermatophyte infections and terbinafine resistance, aligned with WHO priorities on fungal pathogens. The work combines conventional LAMP for taxonomic identification with USS-sbLAMP for SNP-based resistance detection, which is a sensible and innovative approach. I have the following comments:

1. The authors note that the pan-dermatophyte, Microsporum, and T. rubrum assays show non-specific amplification beyond 30 minutes and apply somewhat arbitrary time cutoffs (30 or 35 minutes). This is concerning for a point-of-care assay, particularly because late amplification of Candida albicans by the pan-dermatophyte and T. rubrum assays in the colorimetric format (Figure 3, lines 265-267) is a real specificity problem.

2. Selection of L393F as the sole resistance marker.
While L393F is well justified, terbinafine resistance is also conferred by F397L, F397I, F397V, L335F, H440Y, and others. A single SNP-targeted assay will miss a substantial fraction of resistant isolates, limiting clinical utility. The authors should either include additional SNPs or more clearly frame this as a proof-of-concept and discuss the path to multiplexing.

3. The assays were run on a Bio-Rad CFX96 real-time PCR system-a benchtop laboratory instrument. Claims about "point-of-care" deployment require demonstration on a portable/handheld isothermal device or, at minimum, with the colorimetric readout under field-relevant conditions (ambient temperature, simple heat block). The colorimetric data (Figure 3) is a step in this direction but is qualitative and only tested on four isolates.

4. A LoD of 1000 copies/reaction for the pan-dermatophyte (18S) target is surprisingly poor given that 18S is typically a multi-copy locus. This warrants explanation. Did the authors consider ITS or other multi-copy regions? Comparison of LoD with the published assay by Roumani et al. (2022) from which the primers were adapted would be informative.

5. The text references "Figure 5" for colorimetric data (line 265, 383) but the actual figure is labeled Figure 3. Similarly, Figure 3 is cited for sbLAMP data (lines 287, 289) but the figure is labeled Figure 4. This must be corrected throughout as it currently makes the manuscript difficult to follow.

Spectrum00669-26

Authors have developed a LAMP assay for dermatophyte ID and terbinafine resistance as proof-of-concept experiments. The assay appears to be working, but without enough isolates, it is difficult to assess its potential. Authors should use more isolates, and also include T. mentagrophytes genotypes, especially *Tm*VII and *T. indotineae*, as they are emerging pathogens. Also, assay sensitivity should be assessed with gDNA, not synthetic DNA. Additionally, assay specificity requires several isolates within the species complex to test whether it works. Using only one isolate per complex is not sufficient, especially if this assay will be used for patient care. The authors used only 10 dermatophyte isolates for assay development, which is inadequate.

Line 129 – Authors talk about L393F mutation, but do not talk about mutations L393S and F397L, which appears to be major mutations causing resistance in *T. indotineae*. https://pubmed.ncbi.nlm.nih.gov/38748419/

Line 149 – Please clearly provide whether synthetic or gDNA used for which assay?

Line 224 – Authors have used synthetic DNA to determine the assay limit of detection. It is well known that synthetic DNA provides far greater sensitivity than genomic DNA due to its simpler structure compared to complex gDNA. Authors should use dermatophyte isolate gDNA for this study to better understand their assay potential, as that is what they will receive in the laboratory for both ID and resistance detection.

Line 233 – Why non template control showed amplification beyond 30 min. Please clarify

Line 247 – Unfortunately, 10 isolates are too small a number to determine assay validity. They should increase the number of total dermatophytes, especially for new assay. Also, why TTP values for template DNA (I believe that is control) with different dermatophytes section in Fig. 2 are different? Is template DNA is inhibiting?

Fig. 3. I don’t see any of mentagrophytes or *T. indotineae* in this figure. Considering the emergence of *T. mentagrophytes* genotype VII, and drug-resistant *T. indotineae*, it would be good to have these two at least for part of this assay.

Fig. 6. Are MT-1 or MT-2 is synthetic construct or mutant strain of *T. rubrum*?

Reviewer #1 (Comments for the Author):

Major comments:

Authors have developed a LAMP assay for dermatophyte ID and terbinafine resistance as proof-of-concept experiments. The **assay appears to be working**, but without enough isolates, it is difficult to assess its potential. Authors should use more isolates, and also include T. mentagrophytes genotypes, especially TmVII and T. indotineae, as they are emerging pathogens. Also, assay sensitivity should be assessed with gDNA, **not synthetic DNA**. Additionally, assay specificity requires several isolates within the species complex to test whether it works. Using only one isolate per complex is not sufficient, **especially if this assay will be used for patient care**. The authors used only 10 dermatophyte isolates for assay development, which is inadequate.

> We thank the reviewer for their detailed assessment of our work. Indeed, we align very closely on their thinking. However, we believe there is one key misunderstanding between our viewpoints and that is that our current work sought to establish a pathfinder towards clinical use but was not intended to demonstrate clinical utility currently. Clarifying this distinction is a key focus of the revised paper with many of the comments made above addressed below in detailed point-by-point responses to ensure this message is clearly made. We certainly hope that with further resources we can move the current platform from bench to clinic with the appropriate clinical isolate controls to demonstrate robust diagnosis at patient side. However, this is, we believe, beyond the scope of what is possible within the standard timeframe of a review cycle.

Specific comments:

Line 129 - Authors talk about L393F mutation, but do not talk about mutations L393S and F397L, which appears to be major mutations causing resistance in *T. indotineae*. https://pubmed.ncbi.nlm.nih.gov/38748419/

> At the time the USS-sbLAMP assay was designed, the published literature and publicly available SQLE sequence data were dominated by *Trichophyton rubrum*, while comparatively limited sequence data were available for the recently emerging species *T. indotineae*. Consequently, the multiple sequence alignment used for assay design was heavily influenced by *T. rubrum* derived sequences. In this light, L393F was selected because it was the most prevalent resistance-associated mutation identified in our sequence dataset and was therefore considered the most suitable target for initial, proof-of-concept assay development. Importantly, the framework described here is readily adaptable to alternative resistance-associated SNPs such as those more recently characterised in emerging pathogens.
>
> To clarify this, our manuscript has been updated to better stress this as an initial proof-of-concept, e.g. '*Given the rapidly evolving landscape of dermatophyte antifungal resistance, including the emergence of T. indotineae, this study was designed as a proof-of-concept framework that can be adapted to additional species and resistance-associated mutations as they emerge*' (line 102), and again in the discussion – '*The assay framework described here is readily adaptable to additional resistance-associated mutations and emerging dermatophyte species, supporting future diagnostic development in an evolving antifungal resistance landscape*' (line 420).

Line 149 - Please clearly provide whether synthetic or gDNA used for which assay?

> We thank the reviewer for highlighting this lack of clarity. The LAMP reaction conditions methods section has been updated to more clearly specify the template DNA used in each application - '*1 µL of template DNA (synthetic DNA for assay optimisation and analytical sensitivity testing, or genomic DNA where indicated).*' (line 179).

Line 224 - Authors have used synthetic DNA to determine the assay limit of detection. It is well known that synthetic DNA provides far greater sensitivity than genomic DNA due to its simpler structure compared to complex gDNA. Authors should use dermatophyte isolate gDNA for this study to better understand their assay potential, as that is what they will receive in the laboratory for both ID and resistance detection.

Again stressing that our purpose here was proof-of-concept, analytical sensitivity was assessed using synthetic DNA. This is consistent with previously published LAMP assay development studies published in *Microbiology Spectrum* (https://journals.asm.org/doi/10.1128/spectrum.05222-22). Synthetic DNA was selected as it provides a defined template concentration, enabling accurate determination of assay performance under controlled conditions. It is important to note that determination of a limit of detection using genomic fungal DNA can be complicated by genome copy number variation between isolates of the loci selected. Use of a synthetic DNA therefore enables standardisation of target copy number across experiments. We agree that in the future, clinical will require extensive pre-testing on genomic DNA for precisely this variability reason.

Line 233 - Why non template control showed amplification beyond 30 min. Please clarify

Late amplification observed in non-template controls is a recognised phenomenon in LAMP assays and is generally attributed to non-specific amplification arising from the use of multiple primers and prolonged reaction times. To minimise the impact of such events on assay interpretation, conservative runtime cut-offs were established based on melt curve analysis (example plot provided in **Figure S1**). This approach is consistent with previous LAMP assay development studies.

To clarify this, we have outlined this concept in the following updated statement '*The Pan-dermatophyte, Microsporum and T. rubrum assays variably showed non-specific amplification in the non-template control (H_2O) beyond 30 minutes (example amplification plot and melt curve in Figure S1). As such, in subsequent assays we capped running times of the Pan-dermatophyte assay to 30 minutes and all other assays to 35 minutes, in line with previously developed LAMP assays (Malpartida-Cardenas et al., 2019).*' (Line 272) within text **results**.

Line 247 - Unfortunately, 10 isolates are too small a number to determine assay validity. They should increase the number of total dermatophytes, especially for new assay. Also, why TTP values for template DNA (I believe that is control) with different dermatophytes section in Fig. 2 are different? Is template DNA is inhibiting?

The primary aim of this work was to establish proof-of-concept for the development of taxonomic and resistance-associated LAMP assays using available clinical isolates (as discussed above). Access to dermatophyte isolates was limited during the study period due restrictions on MTAs with private pathology suppliers. In the future, with human ethics approval, it is certainly our aspiration to extend our analysis to many more clinical isolates. To clarify this, the following sentence in the discussion of the manuscript addresses this limitation – '*Notably, validation was performed using a limited number of clinical isolates and extracted genomic DNA rather than primary clinical specimens. Further validation using clinical samples (hair, skin and nails) will be required to define diagnostic performance under routine use and suitable extraction conditions.*' (Line 385).

The synthetic template positive controls shown in **Figure 2** are assay-specific and therefore are not directly comparable between assays. The figure legend has been updated to clarify that assay-specific synthetic templates were used as positive controls- '*Assay specific synthetic templates (black bars) were used as positive controls and H_2O as negative control.*' (Line 570).

Fig. 3. I don't see any of mentagrophytes or T. indotineae in this figure. Considering the emergence of T. mentagrophytes genotype VII, and drug-resistant T. indotineae, it would be good to have these two at least for part of this assay.

As discussed above, no *T. indotineae* clinical isolates were available at the time of assay development. Importantly, *T. indotineae* belongs to the T. mentagrophytes complex, representatives of which were included in **Figure 3**. A synthetic DNA template derived from *T. indotineae* sequence data was also incorporated later during assay development to show compatibility (see **Supplementary Table S2** for gene block data). Future validation with contemporary *T. indotineae* isolates is of course warranted and would be a priority for future work. To clarify this, the manuscript has been updated to more explicitly discuss the applicability of the assay framework to emerging terbinafine-resistant *T. indotineae* strains.

Fig. 6. Are MT-1 or MT-2 is synthetic construct or mutant strain of T. rubrum?

> We thank the reviewer for highlighting this confusing distinction. Regarding **Figure 6**; MT-1 and MT-2 refer to the mutant-specific USS-sbLAMP assays targeting the two nucleotide variants encoding the L393F substitution. In **Figure 6**, both clinical *T. rubrum* isolates were confirmed by SQLE sequencing to possess the wild-type allele. Accordingly, each isolate was tested using both the WT-specific assay and the MT-specific assay, with the delayed amplification observed in the MT-1 and MT-2 reactions consistent with the wild-type genotype. The figure legend has been revised to clarify this distinction, *'Real-time amplification plot of wild-type Trichophyton rubrum clinical isolates with USS-sbLAMP assays. A) WT vs MT-1 reaction, B) WT vs MT-2 reaction. Pink lines = WT reaction, blue lines = MT-1/-2 reaction. Fluorescence is plotted as relative fluorescence units (RFU) versus amplification cycles. Circles denote T. rubrum A clinical isolate, and squares denote T. rubrum B clinical isolate.'* (Line 598).

Reviewer #2 (Comments for the Author):

This manuscript addresses a clinically relevant and timely problem-the need for rapid point-of-care diagnostics for dermatophyte infections and terbinafine resistance, aligned with WHO priorities on fungal pathogens. The work combines conventional LAMP for taxonomic identification with USS-sbLAMP for SNP-based resistance detection, which is **a sensible and innovative approach**.

> We thank the reviewer for their positive appraisal of our work.

I have the following comments:

1. The authors note that the pan-dermatophyte, Microsporum, and T. rubrum assays show non-specific amplification beyond 30 minutes and apply somewhat arbitrary time cutoffs (30 or 35 minutes). This is concerning for a point-of-care assay, particularly because late amplification of Candida albicans by the pan-dermatophyte and T. rubrum assays in the colorimetric format (Figure 3, lines 265-267) is a real specificity problem.

> We thank the reviewer for this comment. We note again that our aim here is not clinical application per se, but a pathfinder towards this. Reiterating comments made above to Reviewer 1, late amplification events are commonly reported during LAMP assay development and assay-specific interpretation thresholds are frequently applied to minimise false-positive results. Similar runtime cut-offs have been employed in previously published LAMP-based diagnostic assays, including those developed by Malpartida-Cardenas et al., (https://journals.asm.org/doi/10.1128/spectrum.05222-22) We have revised the manuscript to more clearly frame these assays as proof-of-concept diagnostics and to acknowledge that further primer optimisation and validation will be required prior to clinical implementation (see above, comment to reviewer 1).

2. Selection of L393F as the sole resistance marker. While L393F is well justified, terbinafine resistance is also conferred by F397L, F397I, F397V, L335F, H440Y, and others. A single SNP-targeted assay will miss a substantial fraction of resistant isolates, limiting clinical utility. The authors should either include additional SNPs or more clearly frame this as a proof-of-concept and discuss the path to multiplexing.

> We are acutely aware of this limitation, however, want to re-iterate that the L393F mutation was selected as a proof-of-concept target based on available sequence data and its established association with terbinafine resistance. We have revised the manuscript to more clearly frame this work as a proof-of-concept and to highlight that the USS-sbLAMP framework can readily be adapted to additional resistance-associated mutations - '*This is particularly relevant for the emerging pathogen T. indotineae, in which alternative SQLE mutations such as L393S and F397L are increasingly reported and represent logical future targets for assay expansion (Ebert et al., 2020).*' (Line 410).

3. The assays were run on a Bio-Rad CFX96 real-time PCR system-a benchtop laboratory instrument. Claims about "point-of-care" deployment require demonstration on a portable/handheld isothermal device or, at minimum, with the colorimetric readout under field-relevant conditions (ambient temperature, simple heat

block). The colorimetric data (Figure 3) is a step in this direction but is qualitative and only tested on four isolates.

This is a fair point. Real-time amplification experiments were performed using a laboratory-based instrument to facilitate assay optimisation and visualisation of amplification kinetics. Compatibility with a simple colorimetric readout was subsequently demonstrated as an initial step toward field deployment but not as a claim of having reached validated point-of-care application. We have revised the manuscript to clarify that these assays represent a foundation for future point-of-care implementation rather than a fully validated point-of-care diagnostic platform.

4. A LoD of 1000 copies/reaction for the pan-dermatophyte (18S) target is surprisingly poor given that 18S is typically a multi-copy locus. This warrants explanation. Did the authors consider ITS or other multi-copy regions? Comparison of LoD with the published assay by Roumani et al. (2022) from which the primers were adapted would be informative.

We thank the reviewer for this astute point. The 18S locus was selected following evaluation of multiple candidate taxonomic markers, including ITS. Although ITS offers the advantage of high copy number, we found it difficult to identify primer-binding regions that were sufficiently conserved across dermatophytes while maintaining discrimination from non-dermatophyte fungi. The 18S locus provided the best balance of inclusivity and specificity for a pan-dermatophyte assay.

Direct comparison with Roumani et al. (2022) is challenging, as analytical sensitivity in that study was reported using genomic DNA concentration, whereas the present study used defined synthetic DNA copy numbers. Given the potential for polymorphism and reproducibility of 18S in our hands, we felt it appropriate to continue with this locus.

5. The text references "**Figure 5**" for colorimetric data (line 265, 383) but the actual figure is **labeled Figure 3**. Similarly, **Figure 3 is cited for sbLAMP data** (lines 287, 289) but the **figure is labeled Figure 4**. This must be corrected throughout as it currently makes the manuscript difficult to follow.

We thank the reviewer for their eagle eyes! We have been through the paper again and all figure citations have been reviewed and corrected throughout the manuscript.

We trust that with these changes made (and highlighted in the manuscript) the work might now be suitable for publication and look forward to your favourable review of our revised manuscript.

Re: Spectrum00669-26R1 (**Development of a Rapid LAMP Assay towards Point-of-Care Detection of Dermatophyte Infections and Antifungal Resistance**)

Dear Prof. Jake Baum:

Thank you for the privilege of reviewing your work. Below you will find my comments, instructions from the Spectrum editorial office, and the reviewer comments.

Thank you for the opportunity for the reviewers and editor to review your revised manuscript.

As described in our earlier direct email correspondence, a re-adjustment of the manuscript as a Resource Report instead of a Research Article was suggested. The senior editor and I have agreed that re-adjustment of this manuscript to a Resource Report is appropriate. Please let us know if you and the co-authors are amenable to this article type adjustment to a Resource Report. We will be able to make this adjustment internally once we receive your approval.

Please see the following minor comments to address before article acceptance:
1. In the Results section under the "Incorporation of USS Primers for Enhancement of sbLAMP", Figure 4 is still being referenced. Was the intention to reference Figure 5 in this Results section? See Lines 342-343 (marked up manuscript pdf) / Lines 306-307 (merged manuscript pdf).
2. Like what is seen in Figure 4, Figure 5 also requires either an incorporated figure key or additional text in the figure legend describing differences between the red bars versus white bars, as well as describing the gray-shaded region and non-shaded region of each panel (mutant template versus WT template, respectively?), A and B.
3. For Line 417 (marked up manuscript pdf) / Line 367 (merged manuscript pdf) in the Discussion section, is it meant to reference Figure 3 instead of Figure 5 here, as it is referencing simple colorimetric readouts?

Please return the manuscript within 60 days; if you cannot complete the modification within this time period, please contact me. If you do not wish to modify the manuscript and prefer to submit it to another journal, notify me immediately so that the manuscript may be formally withdrawn from consideration by Spectrum.

Revision Guidelines
To submit your modified manuscript, log into the submission site at https://spectrum.msubmit.net/cgi-bin/main.plex. Go to Author Tasks and click the appropriate manuscript title to begin. The information you entered when you first submitted the paper will be displayed; update this as necessary. Note the following requirements:

• Upload point-by-point responses to the issues raised by the reviewers in a file named "Response to Reviewers," NOT in your cover letter.
• Upload a compare copy of the manuscript (without figures) as a "Marked-Up Manuscript" file.
• Upload a clean .DOC/.DOCX version of the revised manuscript and remove the previous version.
• Each figure must be uploaded as a separate, editable, high-resolution file (TIFF or EPS preferred), and any multipanel figures must be assembled into one file.
• Any supplemental material intended for posting by ASM should be uploaded with their legends separate from the main manuscript. You can combine all supplemental material into one file (preferred) or split it into a maximum of 10 files with all associated legends included.

For complete guidelines on revision requirements, see our Submission and Review Process webpage. Submission of a paper that does not conform to guidelines may delay acceptance of your manuscript.

Data availability: ASM policy requires that data be available to the public upon online posting of the article, so please verify all links to sequence records, if present, and make sure that each number retrieves the full record of the data. If a new accession number is not linked or a link is broken, provide Spectrum production staff with the correct URL for the record. If the accession numbers for new data are not publicly accessible before the expected online posting of the article, publication may be delayed; please contact production staff (Spectrum@asmusa.org) immediately with the expected release date.

Publication Fees: For information on publication fees and which article types are subject to charges, visit our website. If your manuscript is accepted for publication and any fees apply, you will be contacted separately about payment during the production process; please follow the instructions in that e-mail. Arrangements for payment must be made before your article is published.

ASM Membership: Corresponding authors may join or renew ASM membership to obtain discounts on publication fees. Need to upgrade your membership level? Please contact Customer Service at Service@asmusa.org.

The ASM Journals program strives for constant improvement in our submission and publication process. Please tell us how we

can improve your experience by taking this quick Author Survey.

Thank you for submitting your paper to Spectrum.

Sincerely,
June Chan
Editor
Microbiology Spectrum

Response to reviewers Wunderlich et al., 2026

1. *In the Results section under the "Incorporation of USS Primers for Enhancement of sbLAMP", Figure 4 is still being referenced. Was the intention to reference Figure 5 in this Results section? See Lines 342-343 (marked up manuscript pdf) / Lines 306-307 (merged manuscript pdf).*

Thank you for picking up this error. The text has now been amended to reference 'Figure 5' as intended.

2. *Like what is seen in Figure 4, Figure 5 also requires either an incorporated figure key or additional text in the figure legend describing differences between the red bars versus white bars, as well as describing the grey-shaded region and non-shaded region of each panel (mutant template versus WT template, respectively?), A and B.*

Thank you for highlighting this. The Figure legend for Figure 5 has now been updated to include these additional descriptors and improve figure interpretation:

'**Figure 5. Performance comparison of USS primers with different lengths added to sbLAMP reaction for detection of SQLE mutations.** A) WT vs MT-1 reaction, B) WT vs MT-2 reaction. TTP of specific reactions shown in the lower sections and TTP of unspecific reactions are in the upper section. Red bars represent amplification of the WT template and white bars represent amplification of the corresponding MT template. In each panel, the grey-shaded region shows results obtained using the WT assay, and the unshaded region shows results obtained using the corresponding MT-specific assay. Selected USS primers are indicated with arrows.'

3. *For Line 417 (marked up manuscript pdf) / Line 367 (merged manuscript pdf) in the Discussion section, is it meant to reference Figure 3 instead of Figure 5 here, as it is referencing simple colorimetric readouts?*

Thank you for highlighting this error. The text has now been amended to reference 'Figure 3' as intended.

Re: Spectrum00669-26R2 (**Development of a Rapid LAMP Assay towards Point-of-Care Detection of Dermatophyte Infections and Antifungal Resistance**)

Dear Prof. Jake Baum:

The authors have addressed reviewers' comments adequately, and the manuscript is suitable for acceptance. As discussed, the article type will be adjusted to a Resource Report from a Research Article.

Your manuscript has been accepted, and I am forwarding it to the ASM production staff for publication. Your paper will first be checked to make sure all elements meet the technical requirements. ASM staff will contact you if anything needs to be revised before copyediting and production can begin. Otherwise, you will be notified when your proofs are ready to be viewed.

Data Availability: ASM policy requires that data be available to the public upon online posting of the article, so please verify all links to sequence records, if present, and make sure that each number retrieves the full record of the data. If a new accession number is not linked or a link is broken, provide production staff with the correct URL for the record. If the accession numbers for new data are not publicly accessible before the expected online posting of the article, publication may be delayed; please contact ASM production staff immediately with the expected release date.

Publication Fees: For information on publication fees and which article types have charges, please visit our website. We have partnered with Copyright Clearance Center (CCC) to collect author charges. If fees apply to your paper, you will receive a message from no-reply@copyright.com with further instructions. For questions related to paying charges through RightsLink, please contact CCC at ASM_Support@copyright.com or toll free at +1-877-622-5543. CCC makes every attempt to respond to all emails within 24 hours.

ASM Membership: Corresponding authors may join or renew ASM membership to obtain discounts on publication fees. Need to upgrade your membership level? Please contact Customer Service at Service@asmusa.org.

Embargo Policy: A press release may be issued as soon as the manuscript is posted on the Spectrum Latest Articles webpage. The corresponding author will receive an email with the subject line "ASM Journals Author Services Notification" when the article is available online.

Cover Image Submissions: If you would like to submit a potential Cover Image, please email a file and a short legend to spectrum@asmusa.org. Please note that we can only consider images that (i) the authors created or own and (ii) have not been previously published. By submitting, you agree that the image can be used under the same terms as the published article. Image File requirements: TIF/EPS, 7.5 inches wide by 8.25 inches tall (at least 2,250 pixels wide by 2,475 pixels tall), minimum 300 dpi resolution (600 dpi preferred), RGB, and no figure elements, e.g., arrows or panel labels. The legend should be a short description of the image, 1-2 sentences recommended. Please use the ASM Journals Cover Image Sizing Tool (https://journals.asm.org/pb-assets/html/asm-cover-sizing-tool.html) to ensure that your proposed cover image meets our size requirements.

The ASM Journals program strives for constant improvement in our submission and publication process. Please tell us how we can improve your experience by taking this quick Author Survey.

Thank you for submitting your paper to Spectrum.

Sincerely,
June Chan
Editor
Microbiology Spectrum
