# Supplementary material for: Development of a rapid LAMP assay toward point-of-care detection of dermatophyte infections and antifungal resistance
Source: Microbiol Spectr. 2026 Sep 24;14(11):e00669-26. doi: 10.1128/spectrum.00669-26 (PMC13729109; doi:10.1128/spectrum.00669-26)
Supplement: Supplementary file 2 [file spectrum.00669-26-s0001.docx]

**Development of a Rapid LAMP Assay towards Point-of-Care Detection of Dermatophyte Infections and Antifungal Resistance**

Georgia Wunderlich^1^, Shannen Butterly, Michael Johnson^1^, Artiene Tatian^2,3^* and Jake Baum^1^*

^1^ School of Biomedical Sciences, University of New South Wales, Sydney, Australia.

^2^ School of Clinical Medicine, University of New South Wales, Sydney, Australia.

^3^ Department of Paediatric Dermatology, Sydney Children's Hospital Randwick, Sydney, New South Wales, Australia

*Corresponding author.

For correspondence, email: [artiene.tatian@health.nsw.gov.au](mailto:artiene.tatian@health.nsw.gov.au) or [jake.baum@unsw.edu.au](mailto:jake.baum@unsw.edu.au)

**SUPPLEMENTARY DATA (FIGURE & TABLES)**

**Figure S1. Amplification and melt curve analysis of Pan-dermatophyte LAMP assay.** A) Amplification plot showing relative fluorescence units (RFU) versus cycle number. B) Melt curve analysis of the same reactions displayed as the negative derivative of fluorescence over temperature (-dRFU/dT). Blue traces represent target synthetic template and red traces represent non-template control (H_2_O).

**Table S1. Sequences of all primers used in this manuscript**. For sbLAMP specific primers, selected primers are highlighted in yellow and bold.

| **Assay** | **Gene** | **Source** | **Primer** | **Length (bp)** | **Sequence (5’ – 3’)** |
| --- | --- | --- | --- | --- | --- |
| Pan-dermatophyte | 18S | This study | F3 | 19 | ACGGGGAATTAGGGTTCGA |
|  |  |  | B3 | 19 | TTGGAGCTGGAATTACCGC |
|  |  |  | FIP | 44 | ATTGGGTAATTTGCGCGCCTGTTTTAGAGGGAGCCTGAGAAACG |
|  |  |  | BIP | 44 | TACAGGGCTCTTTAGGGTCTTTGTCTTGCCCTCCAATTGTTCCT |
|  |  |  | LF | 21 | GCCTTCCTTGGATGTGGTAGC |
| Microsporum | Tef1a | This study | F3 | 17 | TGGGATGTGGCTTGGCG |
|  |  |  | B3 | 18 | GGTGATACCACGCTCACG |
|  |  |  | FIP | 40 | TGTGAATTGCGGCTCGATCGTTATCTGCAACCCCCACCAA |
|  |  |  | BIP | 39 | AGGAAGCCGAAGAGTTGGGCCTCAGCCTTGAGCTTGTCA |
|  |  |  | LF | 18 | GTGGTGATGGCGGTGTTG |
|  |  |  | LB | 21 | AGTCCTTCAAGTATGCCTGGG |
| T. rubrum | ITS1 | Seo, Mi-Tan et al 2022 | F3 | 20 | CAAGGTTTCCGTAGGTGAAC |
|  |  |  | B3 | 17 | TCGAGGCTCCCAGAAGG |
|  |  |  | FIP | 45 | GAACGTCGGTCCCTATCGTGGTTTTTGCGGAAGGATCATTAACGC |
|  |  |  | BIP | 39 | GGCCGTACGCCCCCATTCTTTTTACGGCTCTCCCTGGCA |
|  |  |  | LB | 19 | CTACCTCACCCGGTTGCCT |
| T. mentagrophytes | ITS1 | Tang et al., 2022 | F3 | 18 | CCACGATAGGGCCAAACG |
|  |  |  | B3 | 20 | GCTCAGACTGACAGCTCTTC |
|  |  |  | FIP | 44 | CCGAGGCAACCGAGTAAGGTAGTTTTCGTCAGGGGTGAGCAGAT |
|  |  |  | BIP | 43 | TCTTCCAGGAGAGCCGTTCGGTTTTTTTTGCGTCTGTCCTCCG |
|  |  |  | LF | 17 | AAGAATGGGGCGGTACG |
|  |  |  | LB | 21 | GAGCCTCTCTTTAGTGGCTAA |
| sbLAMP (all reactions) | SQLE | This study | F3 | 25 | CTGATTTAAGTGATACCAAACTTGT |
|  |  |  | B3 | 21 | GGGGTCTACATAGTAAAACGT |
|  |  |  | LF | 22 | ACAGAAATCAACGACTTCCGTT |
| sbLAMP (WT TTA) | SQLE | This study | FIP (25) | 44 | TAAGGCCTGAGCAAGAATATTGATG-TGTCCAAATTCCACTGGC |
|  |  |  | FIP (23) | 42 | TAAGGCCTGAGCAAGAATATTGA-TGTCCAAATTCCACTGGC |
|  |  |  | **FIP (21)** | **40** | **TAAGGCCTGAGCAAGAATATT-TGTCCAAATTCCACTGGC** |
|  |  |  | FIP (19) | 38 | TAAGGCCTGAGCAAGAATA-TGTCCAAATTCCACTGGC |
|  |  |  | FIP (17) | 36 | TAAGGCCTGAGCAAGAA-TGTCCAAATTCCACTGGC |
|  |  |  | BIP (27) | 49 | ATACTCTATATTCGCCGCTGGTGGTAA-ACCACTACCCAATAACAAACG |
|  |  |  | BIP (25) | 47 | ATACTCTATATTCGCCGCTGGTGGT-ACCACTACCCAATAACAAACG |
|  |  |  | BIP (23) | 45 | ATACTCTATATTCGCCGCTGGTG-ACCACTACCCAATAACAAACG |
|  |  |  | **BIP (21)** | **43** | **ATACTCTATATTCGCCGCTGG-ACCACTACCCAATAACAAACG** |
|  |  |  | BIP (19) | 41 | ATACTCTATATTCGCCGCT-ACCACTACCCAATAACAAACG |
|  |  |  | BIP (17) | 39 | ATACTCTATATTCGCCG-ACCACTACCCAATAACAAACG |
| sbLAMP (MT1 TTT) | SQLE | This study | FIP (25) | 44 | AAAGGCCTGAGCAAGAATATTGATG-TGTCCAAATTCCACTGGC |
|  |  |  | FIP (23) | 42 | AAAGGCCTGAGCAAGAATATTGA-TGTCCAAATTCCACTGGC |
|  |  |  | FIP (21) | 40 | AAAGGCCTGAGCAAGAATATT-TGTCCAAATTCCACTGGC |
|  |  |  | FIP (19) | 38 | AAAGGCCTGAGCAAGAATA-TGTCCAAATTCCACTGGC |
|  |  |  | **FIP (17)** | **36** | **AAAGGCCTGAGCAAGAA-TGTCCAAATTCCACTGGC** |
|  |  |  | BIP (27) | 49 | TTACTCTATATTCGCCGCTGGTGGTAA-ACCACTACCCAATAACAAACG |
|  |  |  | BIP (25) | 47 | TTACTCTATATTCGCCGCTGGTGGT-ACCACTACCCAATAACAAACG |
|  |  |  | BIP (23) | 45 | TTACTCTATATTCGCCGCTGGTG-ACCACTACCCAATAACAAACG |
|  |  |  | **BIP (21)** | **43** | **TTACTCTATATTCGCCGCTGG-ACCACTACCCAATAACAAACG** |
|  |  |  | BIP (19) | 41 | TTACTCTATATTCGCCGCT-ACCACTACCCAATAACAAACG |
|  |  |  | BIP (17) | 39 | TTACTCTATATTCGCCG-ACCACTACCCAATAACAAACG |
| sbLAMP (MT2 TTC) | SQLE | This study | FIP (25) | 44 | GAAGGCCTGAGCAAGAATATTGATG-TGTCCAAATTCCACTGGC |
|  |  |  | FIP (23) | 42 | GAAGGCCTGAGCAAGAATATTGA-TGTCCAAATTCCACTGGC |
|  |  |  | FIP (21) | 40 | GAAGGCCTGAGCAAGAATATT-TGTCCAAATTCCACTGGC |
|  |  |  | FIP (19) | 38 | GAAGGCCTGAGCAAGAATA-TGTCCAAATTCCACTGGC |
|  |  |  | **FIP (17)** | **36** | **GAAGGCCTGAGCAAGAA-TGTCCAAATTCCACTGGC** |
|  |  |  | BIP (27) | 49 | CTACTCTATATTCGCCGCTGGTGGTAA-ACCACTACCCAATAACAAACG |
|  |  |  | BIP (25) | 47 | CTACTCTATATTCGCCGCTGGTGGT-ACCACTACCCAATAACAAACG |
|  |  |  | BIP (23) | 45 | CTACTCTATATTCGCCGCTGGTG-ACCACTACCCAATAACAAACG |
|  |  |  | **BIP (21)** | **43** | **CTACTCTATATTCGCCGCTGG-ACCACTACCCAATAACAAACG** |
|  |  |  | BIP (19) | 41 | CTACTCTATATTCGCCGCT-ACCACTACCCAATAACAAACG |
|  |  |  | BIP (17) | 39 | CTACTCTATATTCGCCG-ACCACTACCCAATAACAAACG |
| WT (TTA) | SQLE | This study | **BB** | **21** | **TAAGGCCTGAGCAAGAATATT** |
|  |  |  | BB | 23 | TAAGGCCTGAGCAAGAATATTGA |
|  |  |  | BB | 25 | TAAGGCCTGAGCAAGAATATTGATG |
|  |  |  | FB | 23 | ATACTCTATATTCGCCGCTGGTG |
|  |  |  | FB | 25 | ATACTCTATATTCGCCGCTGGTGGT |
|  |  |  | **FB** | **27** | **ATACTCTATATTCGCCGCTGGTGGTAA** |
|  |  |  | FB | 29 | ATACTCTATATTCGCCGCTGGTGGTAAGC |
| MT1 (TTT) | SQLE | This study | BB | 17 | AAAGGCCTGAGCAAGAA |
|  |  |  | BB | 19 | AAAGGCCTGAGCAAGAATA |
|  |  |  | **BB** | **21** | **AAAGGCCTGAGCAAGAATATT** |
|  |  |  | BB | 23 | AAAGGCCTGAGCAAGAATATTGA |
|  |  |  | FB | 23 | TTACTCTATATTCGCCGCTGGTG |
|  |  |  | FB | 25 | TTACTCTATATTCGCCGCTGGTGGT |
|  |  |  | FB | 27 | TTACTCTATATTCGCCGCTGGTGGTAA |
|  |  |  | **FB** | **29** | **TTACTCTATATTCGCCGCTGGTGGTAAGC** |
| MT2 (TTC) | SQLE | This study | BB | 17 | GAAGGCCTGAGCAAGAA |
|  |  |  | BB | 19 | GAAGGCCTGAGCAAGAATA |
|  |  |  | BB | 21 | GAAGGCCTGAGCAAGAATATT |
|  |  |  | **BB** | **23** | **GAAGGCCTGAGCAAGAATATTGA** |
|  |  |  | FB | 23 | CTACTCTATATTCGCCGCTGGTG |
|  |  |  | FB | 25 | CTACTCTATATTCGCCGCTGGTGGT |
|  |  |  | FB | 27 | CTACTCTATATTCGCCGCTGGTGGTAA |
|  |  |  | **FB** | **29** | **CTACTCTATATTCGCCGCTGGTGGTAAGC** |

**Table S2. Sequences of synthetic gene templates used in this manuscript.** For USS-sbLAMP assay associated gene templates, the single nucleotide polymorphism targeted is highlighted in red text.

| **Assay** | **GenBank ID** | **Source organism** | **Sequence (5'-3')** |
| --- | --- | --- | --- |
| SQLE WT allele (TTA) | OM313296 | *Trichophyton rubrum* | CATTTAATGATGTCGTTCTCCTCCGGGATCTGCTTAGTCCGGAGGCCGTACCTGATTTAAGTGATACCAAACTTGTTTTAAAACAGCTGTCCAAATTCCACTGGCAACGGAAGTCGTTGATTTCTGTCATCAATATTCTTGCTCAGGCCTT**A**TACTCTATATTCGCCGCTGGTGGTAAGCTTATACTTTCACGCCGTTTGTTATTGGGTAGTGGTTACTAACGTTTTACTATGTAGACCCCAATTTGAAAGTCCTGCAACGAGGCTGCTTCAGGTATTTCCAACTTGGACT |
| SQLE MT-1 allele (TTT) | OM313304 | *Trichophyton rubrum* | CATTTAATGATGTCGTTCTCCTCCGGGATCTGCTTAGTCCGGAGGCCGTACCTGATTTAAGTGATACCAAACTTGTTTTAAAACAGCTGTCCAAATTCCACTGGCAACGGAAGTCGTTGATTTCTGTCATCAATATTCTTGCTCAGGCCTT**T**TACTCTATATTCGCCGCTGGTGGTAAGCTTATACTTTCACGCCGTTTGTTATTGGGTAGTGGTTACTAACGTTTTACTATGTAGACCCCAATTTGAAAGTCCTGCAACGAGGCTGCTTCAGGTATTTCCAACTTGGACT |
| SQLE MT-2 allele (TTC) | OL415201 | *Trichophyton rubrum* | CATTTAATGATGTCGTTCTCCTCCGGGATCTGCTTAGTCCGGAGGCCGTACCTGATTTAAGTGATACCAAACTTGTTTTAAAACAGCTGTCCAAATTCCACTGGCAACGGAAGTCGTTGATTTCTGTCATCAATATTCTTGCTCAGGCCTT**C**TACTCTATATTCGCCGCTGGTGGTAAGCTTATACTTTCACGCCGTTTGTTATTGGGTAGTGGTTACTAACGTTTTACTATGTAGACCCCAATTTGAAAGTCCTGCAACGAGGCTGCTTCAGGTATTTCCAACTTGGACT |
| Pan-fungal 18S | JX431933 | *Trichophyton rubrum* | CTATCAACTTTCGATGGTAGGATAGTGGCCTACCATGGTGGCAACGGGTAACGGGGAATTAGGGTTCGATTCCGGAGAGGGAGCCTGAGAAACGGCTACCACATCCAAGGAAGGCAGCAGGCGCGCAAATTACCCAATCCCGATACGGGGAGGTAGTGACAATAAATACTGATACAGGGCTCTTTAGGGTCTTGTAATTGGAATGAGTACAATTTAAACCCCTTAACGAGGAACAATTGGAGGGCAAGTCTGGTGCCAGCAGCCGCGGTAATTCCAGCTCCAATAGCGTATATTAAAGTTGTTGCAGTTAAAAAGCTCGTAGTTGAACCTTGGG |
| T. rubrum complex ITS1 | JX431934 | *Trichophyton rubrum* | GGCCGGAAAGTTGGTCAAACTCGGTCATTTAGAGGAAGTAAAAGTCGTAACAAGGTTTCCGTAGGTGAACCTGCGGAAGGATCATTAACGCGCAGGCCGGAGGCTGGCCCCCCACGATAGGGACCGACGTTCCATCAGGGGTGAGCAGACGTGCGCCGGCCGTACGCCCCCATTCTTGTCTACCTCACCCGGTTGCCTCGGCGGGCCGCGCTCCCCCTGCCAGGGAGAGCCGTCCGGCGGGCCCTTCTGGGAGCCTCGAGCCGGACCGCGCCCGCCGGAGGACAGACACCAAGAAAAAATTCTCTGAAGAGCTGTCAGTCTGAGCGTTTAGCAAGCACA |
| Microsporum Tef1a | KM678052 | *Microsporum canis* | ATTGCTTTTCGCCCTTTCAACCTACTATGTGTGCTTTTTTTCTCTGGCGGTGGGATGTGGCTTGGCGCTCGCTTGGGCAGCAAAATCTGCAACCCCCACCAACAACACCGCCATCACCACAGGCAACGATCGAGCCGCAATTCACATTCAGATTTGCTTTTTTCTCTTTTATGCCTTGATGCTAATGAACCATCTATAGGAAGCCGAAGAGTTGGGCAAGAAGTCCTTCAAGTATGCCTGGGTTCTTGACAAGCTCAAGGCTGAGCGTGAGCGTGGTATCACCATTGATATCGCCCTCTGGAAGTTCGAGACCCCCAAGTACATGGTCACCGT |
| T. mentagrophytes complex ITS1 | PX248624.1 | *Trichophyton indotineae* | GCGCAGGCCGGAGGCTGGCCCCCCACGATAGGGCCAAACGTCCGTCAGGGGTGAGCAGATGTGCGCCGGCCGTACCGCCCCATTCTTGTCTACCTTACTCGGTTGCCTCGGCGGGCCGCGCTCTTCCAGGAGAGCCGTTCGGCGAGCCTCTCTTTAGTGGCTAAACGCTGGACCGCGCCCGCCGGAGGACAGACGCAAAAAAATTCTTTCAGAAGAGCTGTCAGTCTGAGCGTTAGCAAGCAAAAATCAGTTA |

**Table S3. Details of non-dermatophyte yeast isolates used in the manuscript to assess assay specificity.** All yeasts provided by Megan Lenardon.

| **Species** | **Provider** | **Reference Code** |
| --- | --- | --- |
| *Candida albicans* | Lab strain | SC5314 |
| *Candida albicans* | Lab strain (bloodstream isolate) | AM2003/0191 |
| *Candida glabrata* | American Type Culture Collection | ATCC2001 |
| *Candida glabrata* | Lab strain (bloodstream isolate) | SCSBB415201 |
| *Candida parapsilosis* | American Type Culture Collection | ATCC22019 |
| *Candida parapsilosis* | Lab strain (bloodstream isolate) | SCSXM67507 |
| *Candida tropicalis* | American Type Culture Collection | ATCC750 |
| *Candida tropicalis* | Lab strain (bloodstream isolate) | SCS501148 |
| *Candida dublinensis* | Lab strain (bloodstream isolate) | SCS171549690 |
| *Candida krusei* | Lab strain (bloodstream isolate) | AMR29-01 |
| *Saccharomyces cerevisiae* | Euroscarf | BY4743 |

**Table S4. Amplification results of sbLAMP assay WT template and MT-1 template.** Mean values of two experiments with each reaction in triplicate 1 x10^5^ copies/ reaction mixture. Selected primers highlighted in yellow.

| **sbLAMP** | | | **WT Template** | | | | | | **MT-1 Template** | | | | | |
| --- | --- | --- | --- | --- | --- | --- | --- | --- | --- | --- | --- | --- | --- | --- |
| **B1c (bp)** | **F1c (bp)** | **Specificity** | **TTP mean (min)** | **SD (min)** | **𝚫TTP (min)** | **SD (min)** | **p-value** | | **TTP mean (min)** | **SD (min)** | **𝚫TTP (min)** | **SD (min)** | **p-value** | |
| **21** | **17** | WT | 13.22 | 0.15 | -2.72 | 2.10 | 0.15 | - | **17.21** | **0.62** | **3.99** | **0.71** | **0.00** | ****** |
|  |  | **MT-1** | 15.94 | 2.10 |  |  |  |  | **13.22** | **0.34** |  |  |  |  |
|  | 19 | WT | 11.33 | 0.38 | -3.98 | 1.34 | 0.03 | * | 15.04 | 0.49 | 2.22 | 0.64 | 0.00 | ** |
|  |  | MT-1 | 15.30 | 1.29 |  |  |  |  | 12.82 | 0.41 |  |  |  |  |
|  | **21** | **WT** | **9.00** | **0.81** | **-4.46** | **1.37** | **0.01** | ****** | 11.39 | 1.42 | -0.18 | 1.69 | 0.86 | - |
|  |  | MT-1 | **13.46** | **1.10** |  |  |  |  | 11.57 | 0.91 |  |  |  |  |
|  | 23 | WT | 10.20 | 0.12 | -2.66 | 0.65 | 0.02 | * | 12.54 | 0.42 | 0.63 | 0.43 | 0.11 | - |
|  |  | MT-1 | 12.86 | 0.63 |  |  |  |  | 11.91 | 0.11 |  |  |  |  |
| 23 | 17 | WT | 12.82 | 1.73 | -5.36 | 1.74 | 0.03 | * | 17.15 | 1.59 | 1.10 | 1.75 | 0.36 | - |
|  |  | MT-1 | 18.18 | 0.25 |  |  |  |  | 16.05 | 0.73 |  |  |  |  |
|  | 19 | WT | 13.10 | 0.23 | -5.90 | 2.44 | 0.05 | - | 16.03 | 0.91 | 0.51 | 1.06 | 0.46 | - |
|  |  | MT-1 | 19.00 | 2.43 |  |  |  |  | 15.52 | 0.54 |  |  |  |  |
|  | 21 | WT | 12.50 | 0.13 | -2.02 | 0.30 | 0.00 | ** | 14.33 | 0.60 | 0.60 | 0.79 | 0.26 | - |
|  |  | MT-1 | 14.52 | 0.27 |  |  |  |  | 13.73 | 0.52 |  |  |  |  |
|  | 23 | WT | 13.35 | 0.15 | -4.00 | 1.05 | 0.02 | * | 15.13 | 1.12 | 0.55 | 1.14 | 0.48 | - |
|  |  | MT-1 | 17.35 | 1.04 |  |  |  |  | 14.58 | 0.18 |  |  |  |  |
| 25 | 17 | WT | 15.73 | 0.79 | -8.88 | 4.16 | 0.06 | - | 18.90 | 1.65 | -0.78 | 1.74 | 0.51 | - |
|  |  | MT-1 | 24.61 | 4.08 |  |  |  |  | 19.68 | 0.55 |  |  |  |  |
|  | 19 | WT | 14.16 | 0.36 | -5.68 | 0.39 | 0.00 | *** | 18.26 | 0.14 | 0.10 | 0.89 | 0.86 | - |
|  |  | MT-1 | 19.84 | 0.16 |  |  |  |  | 18.16 | 0.88 |  |  |  |  |
|  | 21 | WT | 13.48 | 0.05 | -3.65 | 0.66 | 0.01 | * | 15.37 | 0.51 | 0.08 | 0.51 | 0.80 | - |
|  |  | MT-1 | 17.13 | 0.65 |  |  |  |  | 15.29 | 0.02 |  |  |  |  |
|  | 23 | WT | 13.68 | 0.62 | -4.26 | 1.24 | 0.01 | ** | 15.07 | 0.76 | -0.70 | 0.77 | 0.25 | - |
|  |  | MT-1 | 17.94 | 1.08 |  |  |  |  | 15.78 | 0.08 |  |  |  |  |
| 27 | 17 | WT | 16.26 | 1.11 | -10.15 | 3.58 | 0.03 | * | 22.24 | 4.95 | 0.05 | 4.96 | 0.99 | - |
|  |  | MT-1 | 26.41 | 3.40 |  |  |  |  | 22.19 | 0.41 |  |  |  |  |
|  | 19 | WT | 14.80 | 0.54 | -7.31 | 1.98 | 0.02 | * | 18.56 | 0.51 | -1.08 | 0.71 | 0.06 | - |
|  |  | MT-1 | 22.10 | 1.90 |  |  |  |  | 19.64 | 0.49 |  |  |  |  |
|  | 21 | WT | 14.08 | 0.25 | -6.25 | 1.11 | 0.01 | ** | 16.42 | 0.44 | -0.91 | 0.49 | 0.05 | * |
|  |  | MT-1 | 20.33 | 1.08 |  |  |  |  | 17.33 | 0.22 |  |  |  |  |
|  | 23 | WT | 13.91 | 0.27 | -6.76 | 2.41 | 0.04 | * | 15.92 | 0.74 | -2.48 | 0.87 | 0.01 | * |
|  |  | MT-1 | 20.67 | 2.39 |  |  |  |  | 18.40 | 0.47 |  |  |  |  |

**Table S5. Amplification results of sbLAMP assay WT template and MT-2 template.** Mean values of two experiments with each reaction in triplicate 1 x10^5^ copies/ reaction mixture. Selected primers highlighted in yellow.

| **sbLAMP** | | | **WT Template** | | | | | | **MT-2 Template** | | | | | |
| --- | --- | --- | --- | --- | --- | --- | --- | --- | --- | --- | --- | --- | --- | --- |
| **B1c (bp)** | **F1c (bp)** | **Specificity** | **TTP mean (min)** | **SD (min)** | **𝚫TTP (min)** | **SD (min)** | **p-value** | | **TTP mean (min)** | **SD (min)** | **𝚫TTP (min)** | **SD (min)** | **p-value** | |
| **21** | **17** | WT | 14.72 | 0.40 | -10.76 | 5.06 | 0.07 | - | **20.56** | **3.89** | **3.48** | **3.90** | **0.43** | **-** |
|  |  | **MT-2** | 25.47 | 5.04 |  |  |  |  | **17.08** | **0.32** |  |  |  |  |
|  | 19 | WT | 11.36 | 0.76 | -9.77 | 4.17 | 0.05 | * | 15.42 | 1.65 | 0.51 | 1.67 | 0.65 | - |
|  |  | MT-2 | 21.13 | 4.10 |  |  |  |  | 14.91 | 0.29 |  |  |  |  |
|  | **21** | **WT** | **11.28** | **0.13** | **-8.23** | **2.84** | **0.04** | ***** | 13.07 | 0.13 | -0.64 | 1.52 | 0.54 | - |
|  |  | MT-2 | **19.51** | **2.84** |  |  |  |  | 13.70 | 1.51 |  |  |  |  |
|  | 23 | WT | 11.24 | 0.55 | -5.22 | 0.90 | 0.00 | *** | 13.65 | 0.52 | -0.66 | 0.94 | 0.30 | - |
|  |  | MT-2 | 16.47 | 0.71 |  |  |  |  | 14.31 | 0.78 |  |  |  |  |
| 23 | 17 | WT | 15.48 | 0.10 | -14.36 | 4.09 | 0.03 | * | 22.21 | 3.18 | 2.20 | 3.33 | 0.35 | - |
|  |  | MT-2 | 29.84 | 4.09 |  |  |  |  | 20.01 | 1.00 |  |  |  |  |
|  | 19 | WT | 14.71 | 0.91 | -11.00 | 2.10 | 0.00 | ** | 15.39 | 0.09 | -3.31 | 0.59 | 0.01 | ** |
|  |  | MT-2 | 25.71 | 1.90 |  |  |  |  | 18.69 | 0.58 |  |  |  |  |
|  | 21 | WT | 12.26 | 0.74 | -11.75 | 3.02 | 0.02 | * | 17.10 | 2.20 | 1.39 | 2.28 | 0.39 | - |
|  |  | MT-2 | 24.01 | 2.93 |  |  |  |  | 15.71 | 0.61 |  |  |  |  |
|  | 23 | WT | 14.38 | 0.22 | -9.64 | 0.82 | 0.00 | ** | 16.92 | 1.11 | -1.53 | 1.19 | 0.13 | - |
|  |  | MT-2 | 24.02 | 0.78 |  |  |  |  | 18.45 | 0.41 |  |  |  |  |
| 25 | 17 | WT | 15.33 | 1.13 | -17.46 | 4.86 | 0.02 | * | 27.75 | 12.58 | 6.26 | 12.66 | 0.48 | - |
|  |  | MT-2 | 32.79 | 4.73 |  |  |  |  | 21.49 | 1.38 |  |  |  |  |
|  | 19 | WT | 15.49 | 0.51 | -10.25 | 0.91 | 0.00 | *** | 15.72 | 1.97 | -7.99 | 5.93 | 0.12 | - |
|  |  | MT-2 | 25.74 | 0.76 |  |  |  |  | 23.71 | 5.60 |  |  |  |  |
|  | 21 | WT | 13.59 | 0.51 | -9.74 | 2.96 | 0.03 | * | 15.52 | 0.77 | -2.12 | 0.91 | 0.02 | * |
|  |  | MT-2 | 23.34 | 2.91 |  |  |  |  | 17.64 | 0.49 |  |  |  |  |
|  | 23 | WT | 14.23 | 0.23 | -10.40 | 2.68 | 0.02 | * | 15.00 | 1.02 | -4.46 | 1.19 | 0.01 | ** |
|  |  | MT-2 | 24.63 | 2.67 |  |  |  |  | 19.46 | 0.61 |  |  |  |  |
| 27 | 17 | WT | 0.00 | 0.00 | -33.86 | 6.31 | 0.01 | * | 21.13 | 3.59 | -2.06 | 3.72 | 0.43 | - |
|  |  | MT-2 | 33.86 | 6.31 |  |  |  |  | 23.19 | 0.96 |  |  |  |  |
|  | 19 | WT | 16.36 | 0.70 | -2.30 | 16.21 | 0.83 | - | 29.31 | 12.37 | 8.17 | 12.39 | 0.37 | - |
|  |  | MT-2 | 18.65 | 16.19 |  |  |  |  | 21.14 | 0.81 |  |  |  |  |
|  | 21 | WT | 14.15 | 0.35 | -7.00 | 1.75 | 0.02 | * | 21.38 | 4.12 | 2.60 | 4.37 | 0.39 | - |
|  |  | MT-2 | 21.15 | 1.72 |  |  |  |  | 18.78 | 1.44 |  |  |  |  |
|  | 23 | WT | 15.66 | 0.30 | -15.65 | 8.05 | 0.08 | - | 21.20 | 3.38 | -0.12 | 3.68 | 0.96 | - |
|  |  | MT-2 | 31.30 | 8.04 |  |  |  |  | 21.31 | 1.45 |  |  |  |  |

**Table S6. USS-sbLAMP amplification results of WT template and MT-1 template.** Mean values of two experiments with each reaction in triplicate 1 x10^5^ copies/ reaction mixture. Selected USS primers highlighted in yellow.

| **sbLAMP_WT_ USS_MT-1_** | | **WT Template** | | **MT-1 Template** | |
| --- | --- | --- | --- | --- | --- |
| **BB length (bp)** | **FB length (bp)** | **TTP mean (min)** | **SD (min)** | **TTP mean (min)** | **SD (min)** |
| 17 | 23 | 11.24 | 0.83 | 15.02 | 1.4 |
|  | 25 | 10.48 | 0.5 | 13.48 | 1.52 |
|  | 27 | 10.73 | 0.54 | 13.89 | 0.66 |
|  | 29 | 11 | 1.16 | 14.27 | 2.44 |
| 19 | 23 | 10.86 | 1.08 | 13.61 | 1.31 |
|  | 25 | 11.48 | 0.5 | 14.6 | 1.45 |
|  | 27 | 11.06 | 0.94 | 12.92 | 1.2 |
|  | 29 | 11.2 | 0.32 | 14.47 | 2.93 |
| **21** | 23 | 11.25 | 0.62 | 14.11 | 1.67 |
|  | 25 | 10.66 | 0.61 | 13.65 | 0.69 |
|  | 27 | 10.81 | 0.6 | 16.02 | 6.1 |
|  | **29** | **9.28** | **0.5** | **16.06** | **5.58** |
| 23 | 23 | 10.31 | 0.73 | 13.28 | 0.87 |
|  | 25 | 10.29 | 1.07 | 13.01 | 0.28 |
|  | 27 | 10.87 | 0.55 | 15.52 | 2.56 |
|  | 29 | 10.76 | 0.58 | 15.04 | 3.51 |
| **sbLAMP_MT-1_ USS_WT_** | | **WT Template** | | **MT-1 Template** | |
| **BB length (bp)** | **FB length (bp)** | **TTP mean (min)** | **SD (min)** | **TTP mean (min)** | **SD (min)** |
| 21 | 23 | 16.46 | 0.81 | 15.33 | 0.86 |
|  | 25 | 15.38 | 0.44 | 14.34 | 1.45 |
|  | 27 | 18.54 | 3.76 | 14.25 | 0.11 |
|  | 29 | 18.06 | 4.2 | 14.98 | 0.22 |
| 23 | 23 | 19.09 | 3.11 | 12.04 | 0.65 |
|  | 25 | 17.07 | 2.69 | 12.32 | 0.6 |
|  | 27 | 21.9 | 5.03 | 12.65 | 0.63 |
|  | 29 | 17.48 | 0.38 | 12.29 | 0.65 |
| **25** | **23** | **30.42** | **6.45** | **14.53** | **0.92** |
|  | 25 | 20.44 | 3.29 | 13.8 | 0.24 |
|  | 27 | 22.26 | 0.03 | 13.56 | 0.48 |
|  | 29 | 16.46 | 2.47 | 13.83 | 0.52 |

**Table S7. S-sbLAMP amplification results of WT template and MT-2 template.** Mean values of two experiments with each reaction in triplicate 1 x10^5^ copies/ reaction mixture. Selected USS primers highlighted in yellow.

| **sbLAMP_WT_ USS_MT-2_** | | **WT Template** | | **MT-2 Template** | |
| --- | --- | --- | --- | --- | --- |
| **BB length (bp)** | **FB length (bp)** | **TTP mean (min)** | **SD (min)** | **TTP mean (min)** | **SD (min)** |
| 17 | 23 | 14.87 | 1.53 | 13.87 | 0.59 |
|  | 25 | 14.99 | 1.08 | 13.57 | 0.91 |
|  | 27 | 14 | 2.13 | 13.87 | 0.27 |
|  | 29 | 13.71 | 0.62 | 13.49 | 0.29 |
| 19 | 23 | 13.44 | 0.11 | 14.13 | 1.21 |
|  | 25 | 13.89 | 0.42 | 14.35 | 0.22 |
|  | 27 | 14.25 | 0.02 | 13.44 | 0.51 |
|  | 29 | 13.87 | 0.31 | 14 | 1.29 |
| **21** | 23 | 14.3 | 0.61 | 12.93 | 0.61 |
|  | 25 | 14.14 | 1.01 | 15.37 | 1.64 |
|  | **27** | **13.49** | **0.59** | **14.92** | **1.31** |
|  | 29 | 12.68 | 0.2 | 13.51 | 0.35 |
| 23 | 23 | 13.85 | 0.82 | 13.81 | 2.54 |
|  | 25 | 14.34 | 0.77 | 13.89 | 3.32 |
|  | 27 | 15.34 | 0.34 | 13.36 | 1.39 |
|  | 29 | 15.57 | 1.38 | 12.81 | 0.46 |
| **sbLAMP_MT-2_ USS_WT_** | | **WT Template** | | **MT-2 Template** | |
| **BB length (bp)** | **FB length (bp)** | **TTP mean (min)** | **SD (min)** | **TTP mean (min)** | **SD (min)** |
| 21 | 23 | 24.5 | N/A | 16.87 | 0.59 |
|  | 25 | 47.67 | N/A | 17.76 | 0.87 |
|  | 27 | 47.63 | N/A | 16.75 | 0.59 |
|  | 29 | 40.77 | N/A | 16.5 | 0.46 |
| **23** | 23 | 43.42 | N/A | 17.86 | 1.26 |
|  | 25 | 31.37 | 4.21 | 16.91 | 1.37 |
|  | 27 | 27.45 | N/A | 15.82 | 0.46 |
|  | **29** | **42.41** | **9** | **15.94** | **0.9** |
| 25 | 23 | 35.19 | 9.37 | 17.06 | 0.44 |
|  | 25 | 43.78 | N/A | 16.53 | 0.43 |
|  | 27 | 28.03 | N/A | 16.19 | 0.71 |
|  | 29 | 31.18 | 4.85 | 15.96 | 0.79 |
|  |  |  | N/A - (1 rep detected) | |  |
